# Supplementary material for: Climate change may induce connectivity loss and mountaintop extinction in Central American forests
Source: Commun Biol. 2021 Jul 15;4:869. doi: 10.1038/s42003-021-02359-9 (PMC8282624; doi:10.1038/s42003-021-02359-9)
Supplement: Supplementary file 2 — Supplementary Information [file 42003_2021_2359_MOESM2_ESM.pdf]

Supporting Figures and Tables

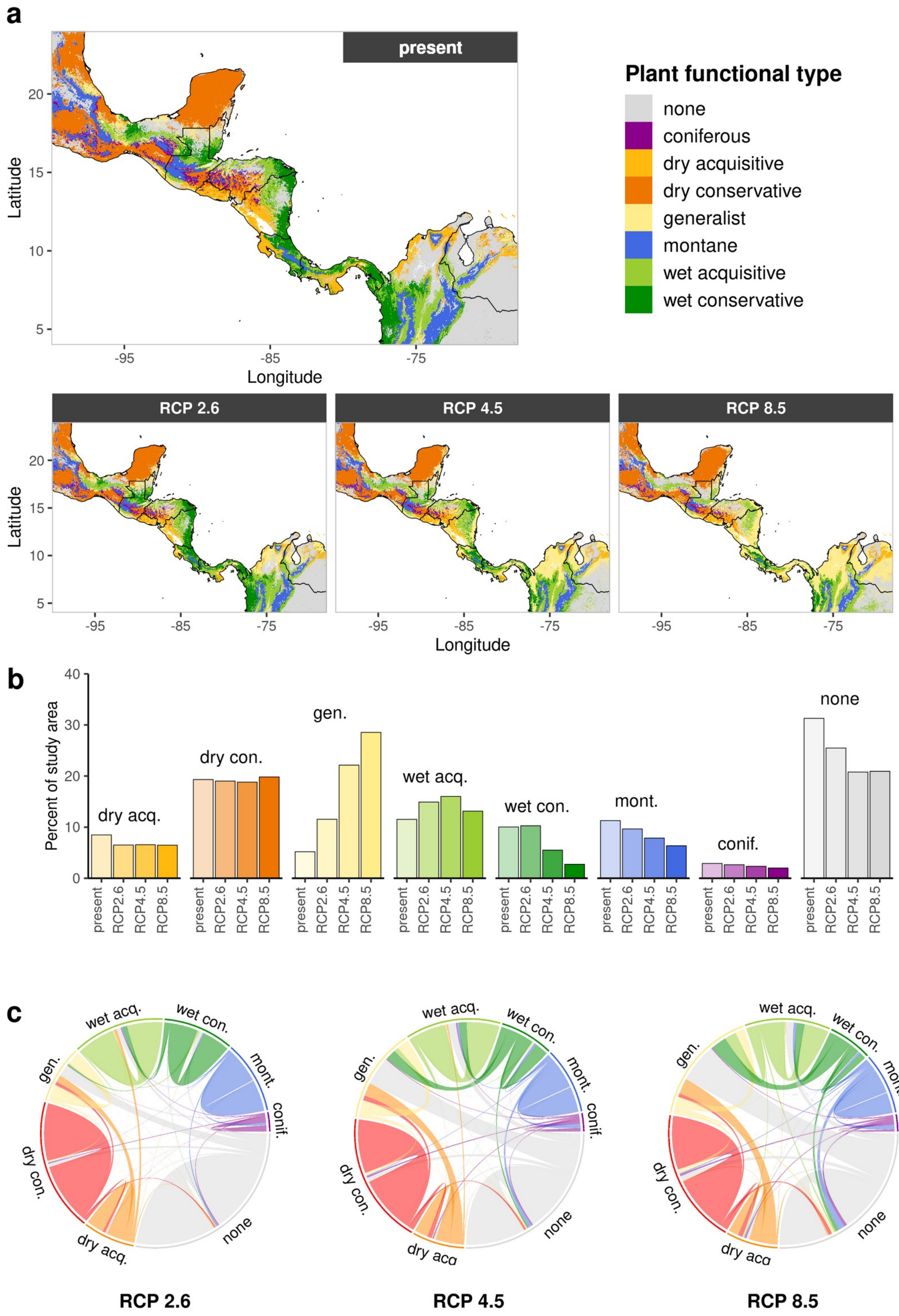

Figure S1: Dominant plant functional types. Model projections for the HadGEM2-AO scenarios. For detailed explanations of the panels see Figure 1.

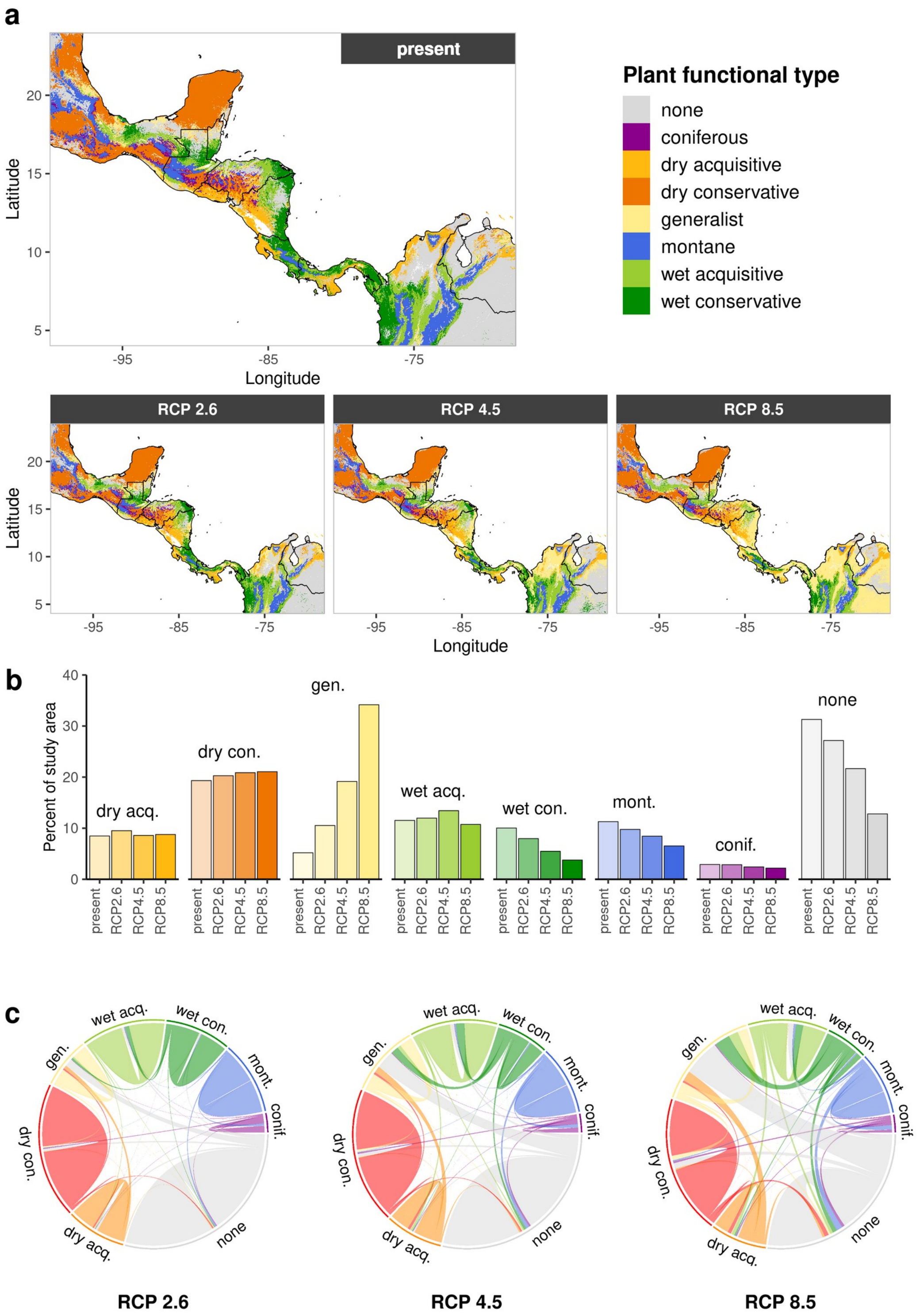

Figure S2: Dominant plant functional types. Model projections for the MPI-ESM-LR scenarios. For detailed explanations of the panels see Figure 1.

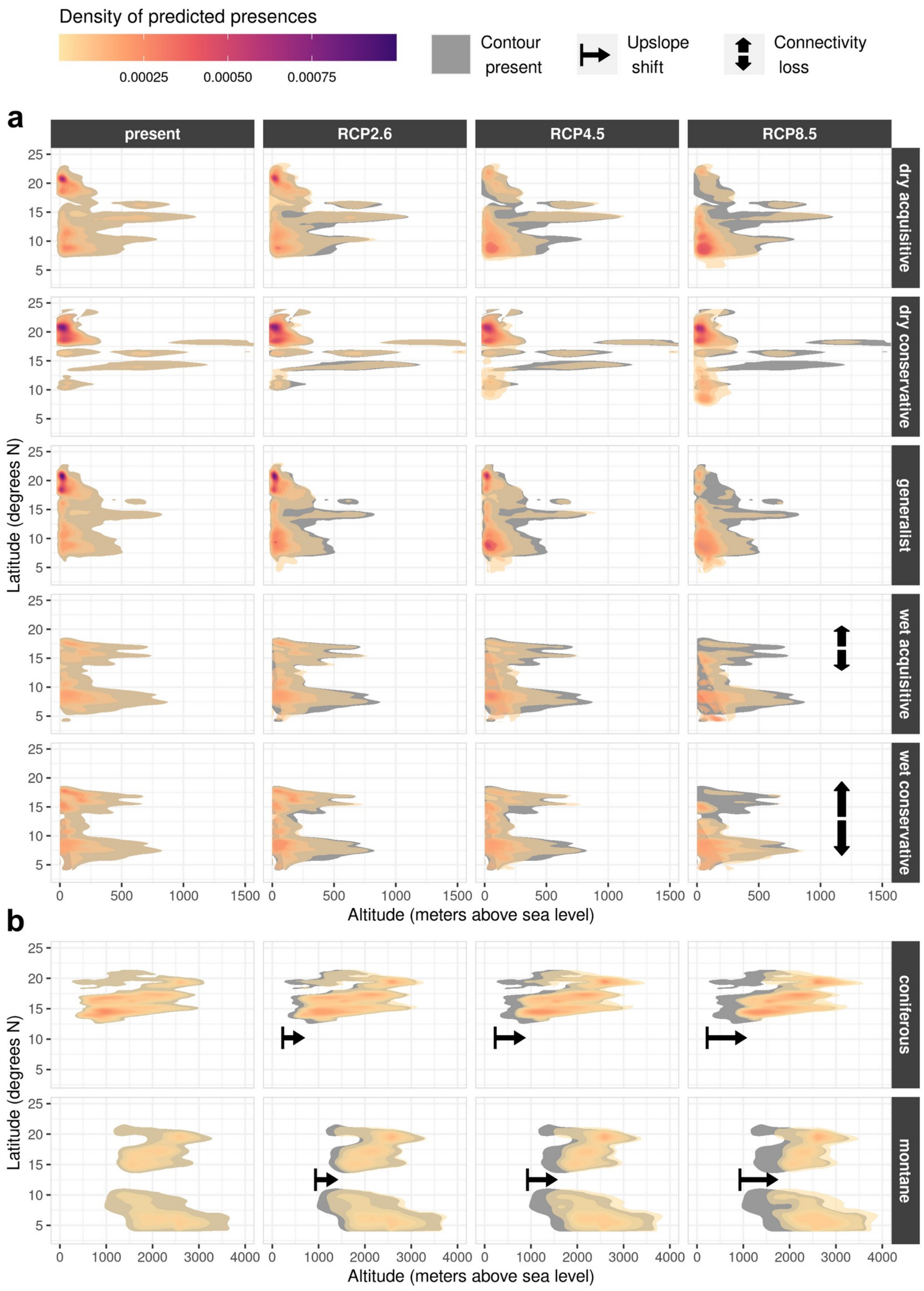

Figure S3: Latitude-altitude suitability shifts. Density plot of projected plant functional type presences (bS-SDM ≥2) across latitudinal and altitudinal gradients for the HadGEM2-AO scenarios. For detailed explanations see Figure 2.

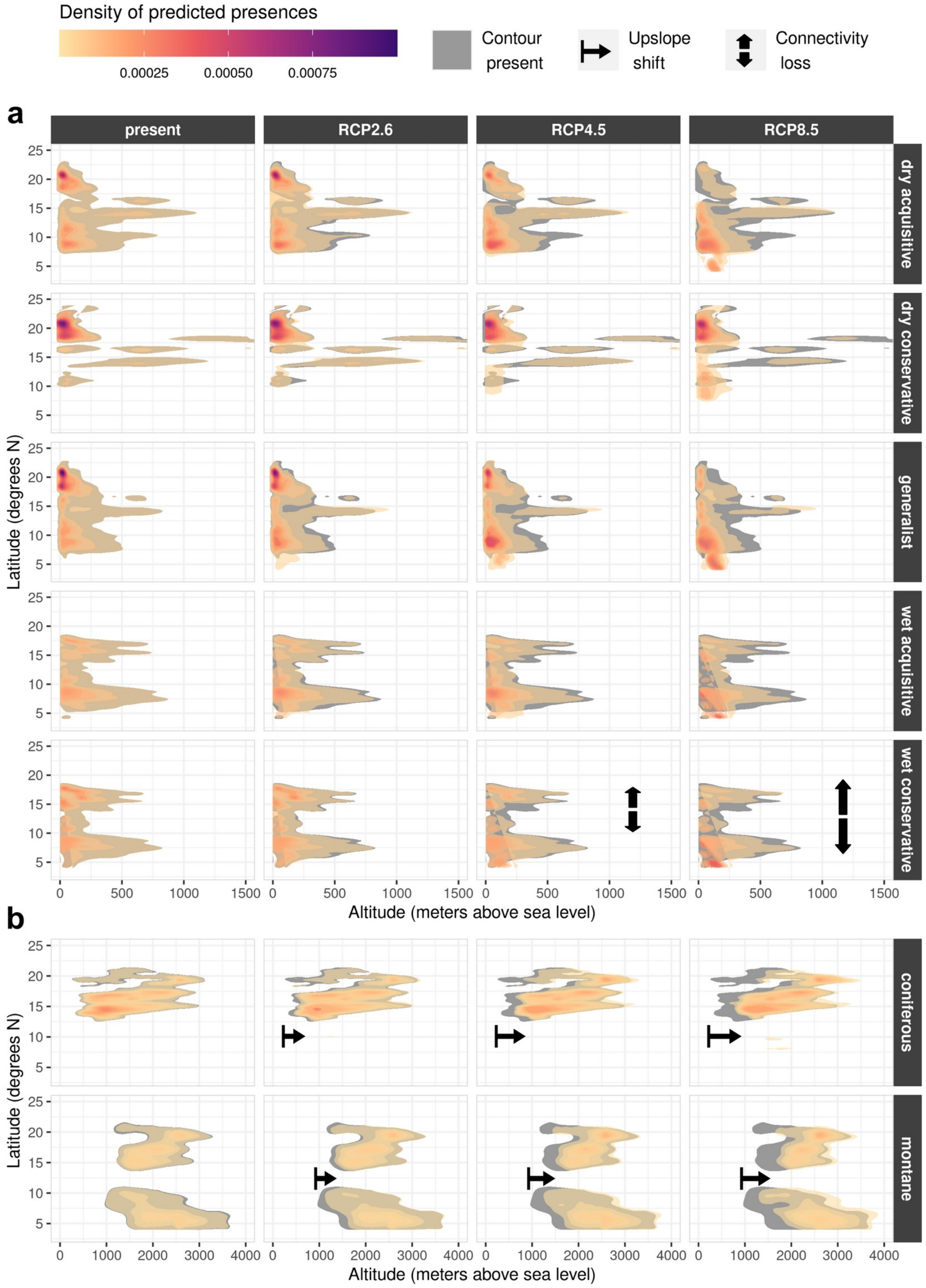

Figure S4: Latitude-altitude suitability shifts. Density plot of projected plant functional type presences (bS-SDM ≥2) across latitudinal and altitudinal gradients for the HadGEM2-AO scenarios. For detailed explanations see Figure 2.

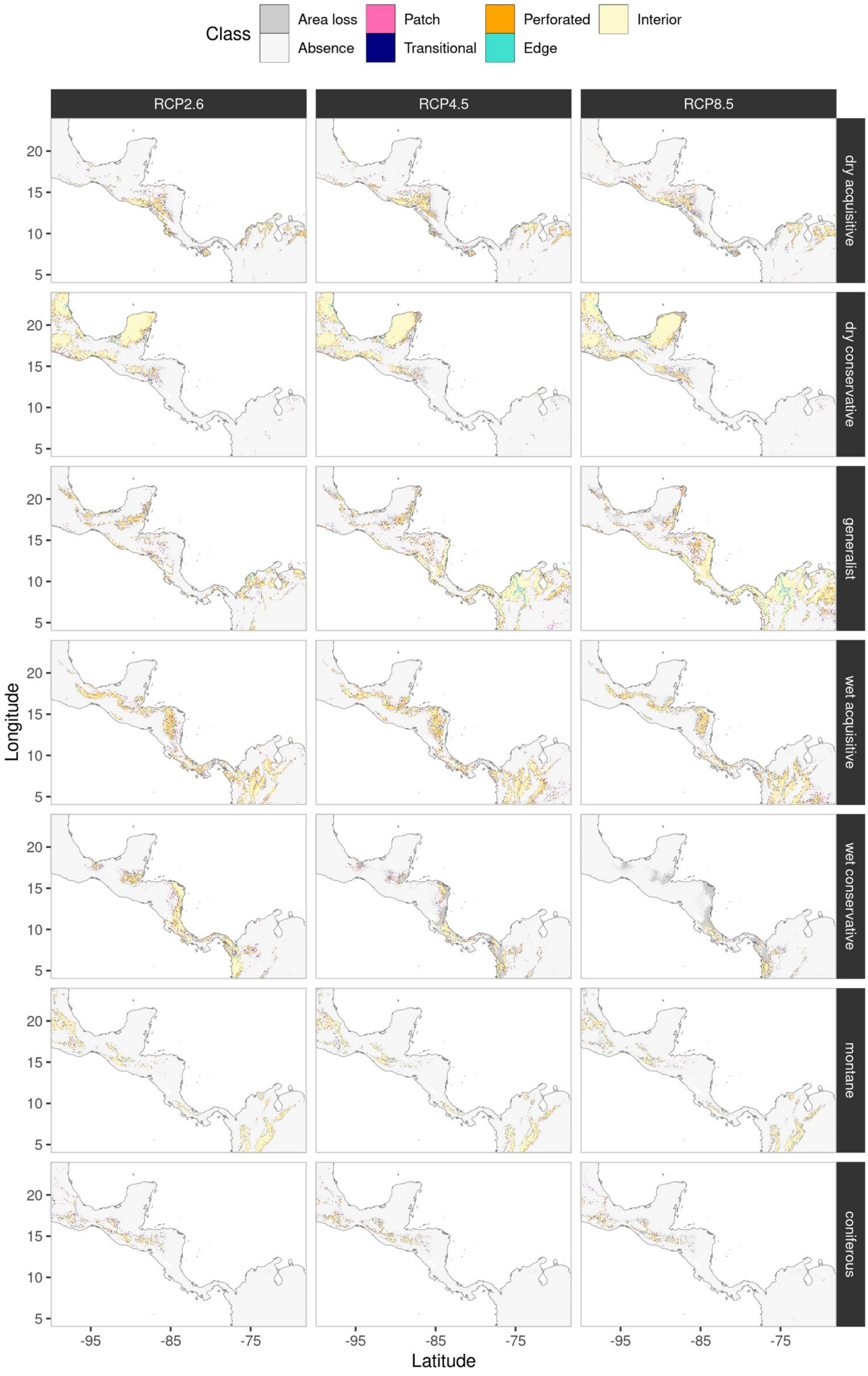

Figure S5: Dominant plant functional type (PFT) fragmentation for the HadGEM2-AO scenarios. Area loss indicates areas which were lost in comparison to present-day projections. Absence indicates, that the respective PFT was not dominant. For other class definitions see Methods.

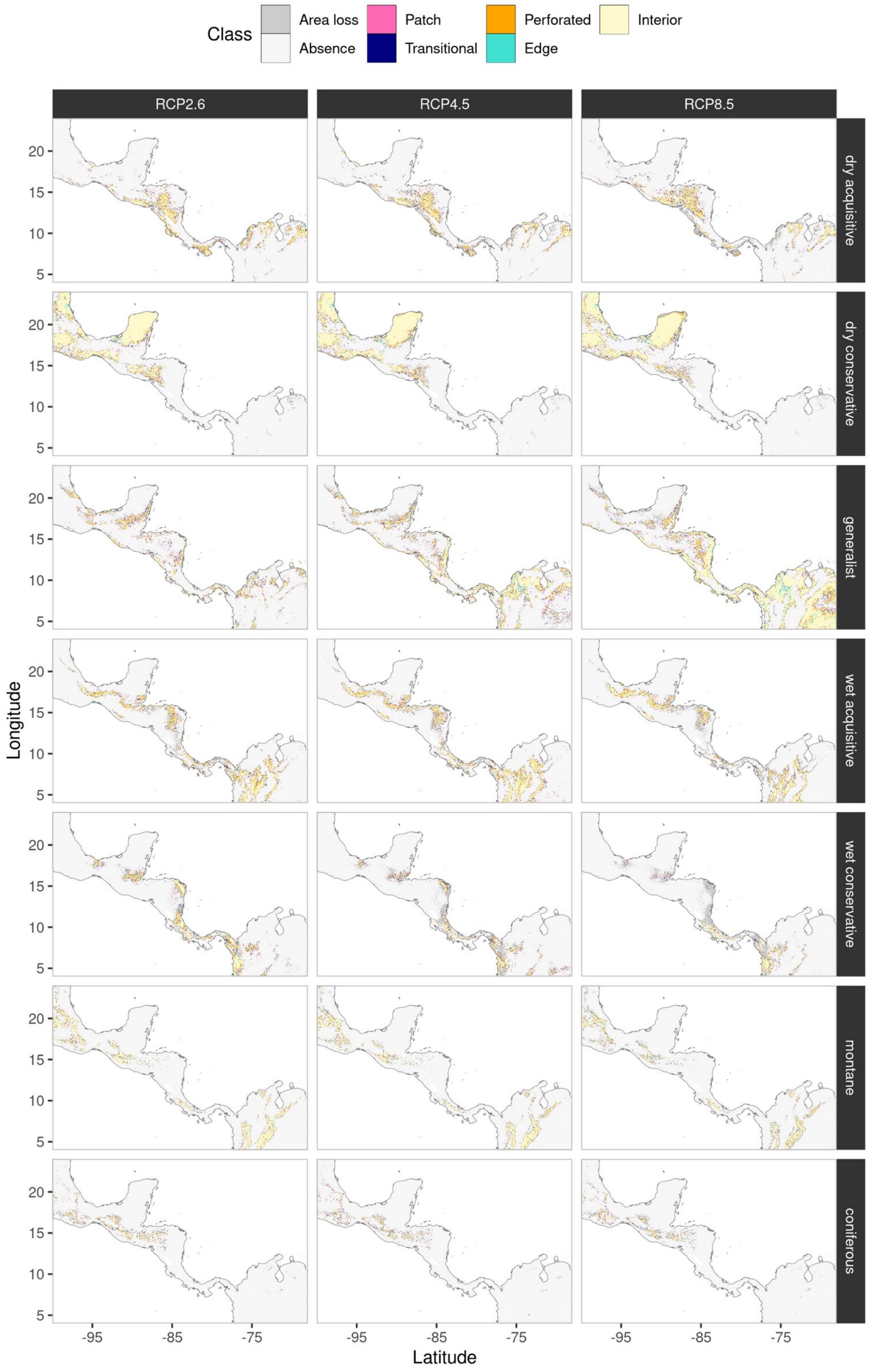

Figure S6: Dominant plant functional type (PFT) fragmentation for the MPI-ESM-LR scenarios. Area loss indicates areas which were lost in comparison to present-day projections. Absence indicates, that the respective PFT was not dominant. For other class definitions see Methods.

Table S1: Dominant PFT area statistics. Column 1 shows shares (%) of the study area covered by each respective plant functional type (PFT) for the present-day projections (in italics) and changes to this number for each climate change scenario (cc=CCSM4, ha=HadGEM2-AO, mp=MPI-ESM-LR). Columns 2-6 again show how much each fragmentation class contributed to the area shares of Column 1.

		Study area share [%]			Patch fraction [%]			Transitional fraction [%]			Perforated fraction [%]			Edge fraction [%]			Interior fraction [%]		
PFT	scenario	*cc*	*ha*	*mp*	*cc*	*ha*	*mp*	*cc*	*ha*	*mp*	*cc*	*ha*	*mp*	*cc*	*ha*	*mp*	*cc*	*ha*	*mp*
Dry acq.	present	8.48			5.80			11.08			27.38			5.27			50.47		
	RCP2.6	+0.35	-1.98	+1.03	6.22	7.68	6.32	11.40	13.77	11.35	28.71	31.01	29.30	4.84	5.02	4.48	48.83	42.52	48.56
	RCP4.5	-0.29	-1.92	+0.10	6.73	6.91	6.23	12.38	13.08	11.61	31.36	31.36	30.79	4.64	4.52	4.28	44.90	44.14	47.10
	RCP8.5	-0.73	-2.01	+0.29	7.73	8.35	6.88	14.26	14.70	12.53	32.33	31.15	29.86	4.53	4.67	4.25	41.16	41.14	46.49
Dry con.	present	19.32			2.10			4.70			11.76			3.61			77.83		
	RCP2.6	+0.36	-0.30	+0.95	2.15	2.05	1.92	4.69	4.33	4.52	11.86	10.83	11.39	3.56	3.60	3.76	77.73	79.19	78.42
	RCP4.5	+0.58	-0.51	+1.55	2.06	2.06	2.01	4.48	4.31	4.36	11.13	10.44	10.62	3.49	3.48	3.59	78.83	79.71	79.41
	RCP8.5	+1.48	+0.50	+1.73	2.28	2.22	2.05	4.79	4.66	4.44	11.92	11.42	10.85	3.58	3.49	3.58	77.43	78.22	79.09
Generalist	present	5.19			14.60			19.36			32.35			7.66			26.03		
	RCP2.6	+4.12	+6.35	+5.34	10.73	8.31	10.54	16.30	14.06	15.89	28.99	27.83	28.81	8.49	8.09	8.88	35.49	41.72	35.89
	RCP4.5	+10.12	+16.95	+13.96	7.31	4.87	7.12	11.87	8.80	11.61	22.20	17.93	22.30	9.05	8.15	8.42	49.57	60.26	50.55
	RCP8.5	+20.01	+23.36	+28.98	5.02	3.74	3.09	8.73	7.13	6.16	17.00	15.12	14.83	7.40	6.74	6.34	61.85	67.26	69.58
Wet acq.	present	11.52			5.55			11.94			32.16			4.78			45.57		
	RCP2.6	+1.21	+3.39	+0.45	5.25	4.78	5.35	11.36	10.83	11.76	31.16	30.94	32.04	4.66	4.49	4.72	47.56	48.95	46.14
	RCP4.5	+2.22	+4.50	+1.91	4.40	4.88	4.70	10.21	10.67	10.65	28.94	29.53	29.21	4.37	4.19	4.40	52.07	50.74	51.04
	RCP8.5	-0.02	+1.62	-0.78	5.21	4.65	4.41	10.41	10.76	10.48	27.86	28.28	28.53	4.39	4.49	4.57	52.14	51.82	52.00
Wet con.	present	10.04			5.79			9.34			22.15			5.70			57.02		
	RCP2.6	-1.11	+0.24	-2.07	6.84	6.40	8.41	10.53	9.61	11.41	23.58	21.51	24.42	5.48	5.35	5.46	53.57	57.12	50.29
	RCP4.5	-5.13	-4.54	-4.56	11.00	10.80	11.37	14.76	14.96	15.54	27.85	26.66	28.87	5.77	5.37	5.68	40.62	42.22	38.54
	RCP8.5	-7.88	-7.31	-6.27	12.50	8.00	9.16	16.78	13.31	12.90	27.38	25.76	24.64	7.02	6.57	6.01	36.31	46.37	47.29

Table S1 continued.

		Study area share [%]			Patch fraction [%]			Transitional fraction [%]			Perforated fraction [%]			Edge fraction [%]			Interior fraction [%]		
PFT	scenario	*cc*	*ha*	*mp*	*cc*	*ha*	*mp*	*cc*	*ha*	*mp*	*cc*	*ha*	*mp*	*cc*	*ha*	*mp*	*cc*	*ha*	*mp*
Montane	present	11.28			1.91			6.44			16.56			3.40			71.69		
	RCP2.6	-1.39	-1.64	-1.53	2.07	2.05	2.03	6.55	6.62	6.75	16.39	16.71	17.15	3.31	3.41	3.56	71.69	71.20	70.50
	RCP4.5	-2.66	-3.42	-2.85	2.09	2.05	2.06	6.62	6.66	6.81	16.81	16.86	17.75	3.48	3.59	3.63	70.99	70.83	69.75
	RCP8.5	-4.49	-4.93	-4.75	2.06	2.10	2.14	7.16	7.02	7.18	18.11	18.17	18.41	3.76	3.77	3.89	68.91	68.95	68.37
Coniferous	present	2.89			11.78			21.18			34.65			6.79			25.59		
	RCP2.6	-0.25	-0.25	-0.04	11.59	11.73	11.63	21.81	21.46	21.06	34.56	34.06	34.21	6.58	6.63	6.51	25.47	26.13	26.59
	RCP4.5	-0.43	-0.56	-0.47	10.97	10.12	12.56	20.75	20.70	22.42	34.86	35.12	33.35	6.80	6.93	6.45	26.60	27.14	25.22
	RCP8.5	-0.71	-0.89	-0.70	10.83	10.79	12.24	21.03	20.38	22.02	34.50	34.52	33.65	6.70	6.68	6.61	26.94	27.63	25.48

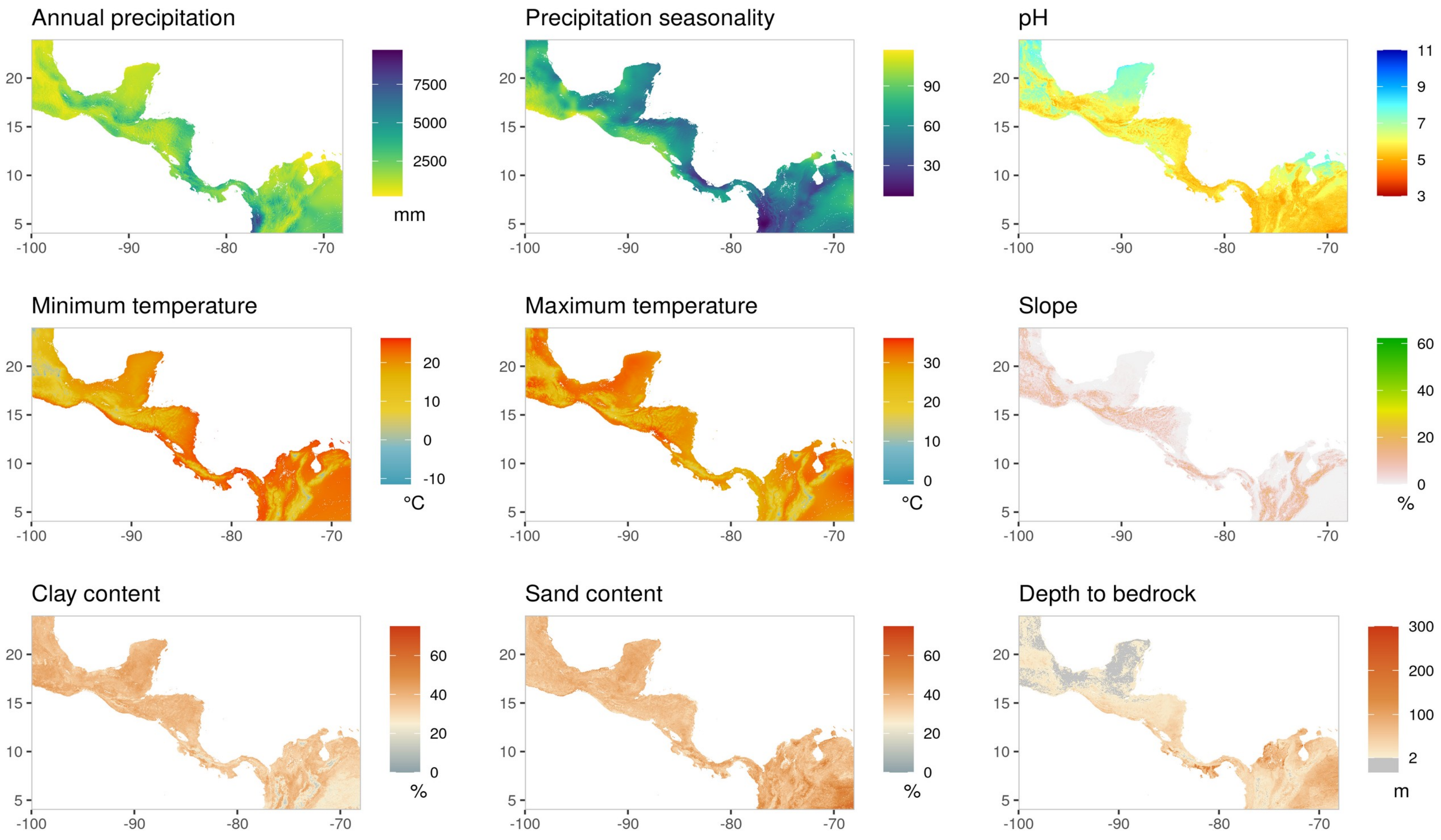

Figure S7: Maps of environmental predictors used for model training.

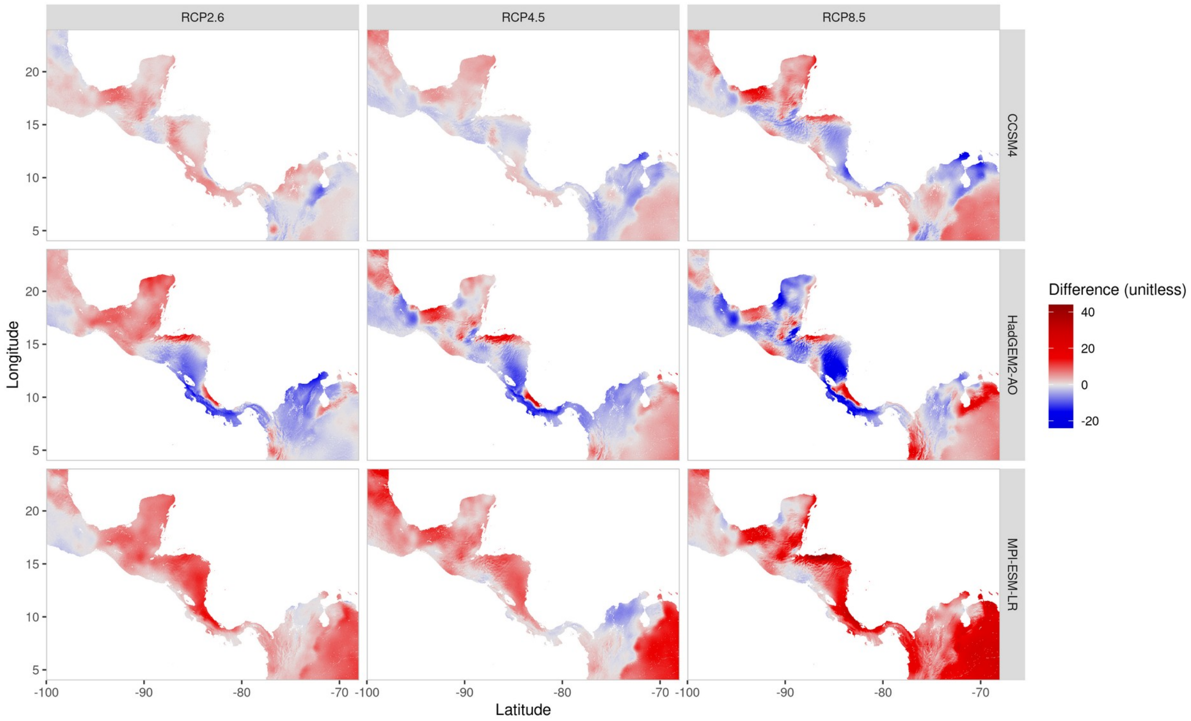

Figure S8: Difference of precipitation seasonality between present-day and climate projections.

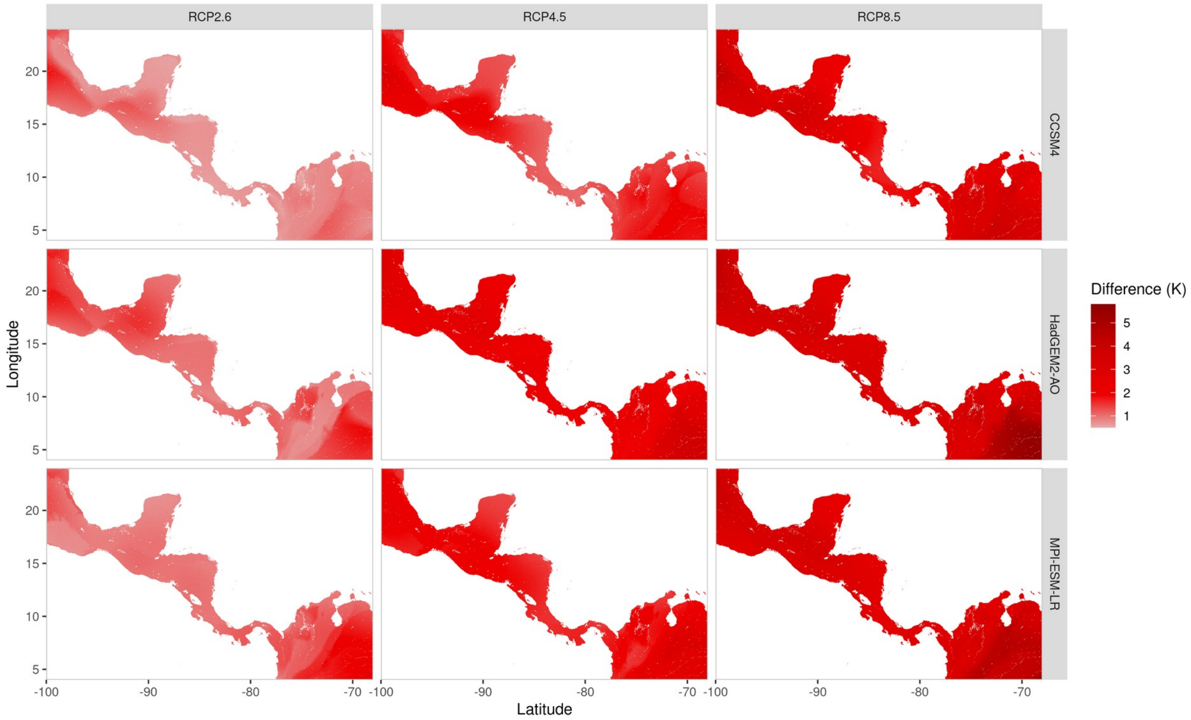

Figure S9: Difference of maximum temperatures between present-day and climate projections.

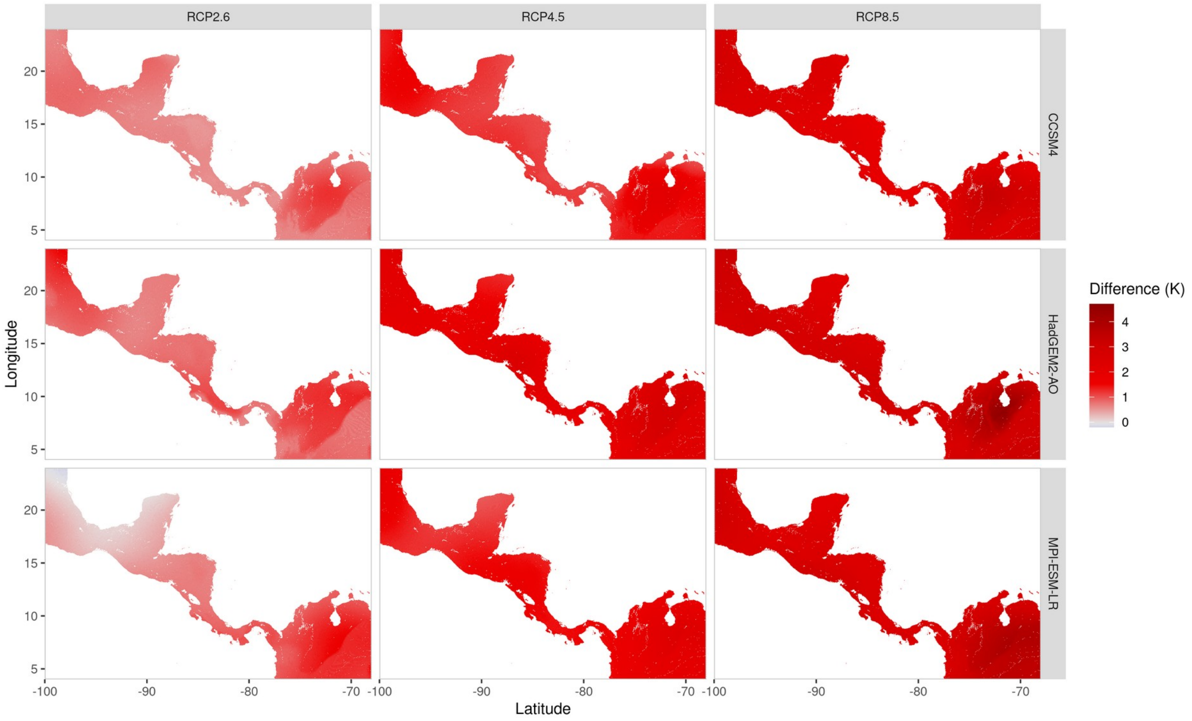

Figure S10: Difference of minimum temperatures between present-day and climate projections.

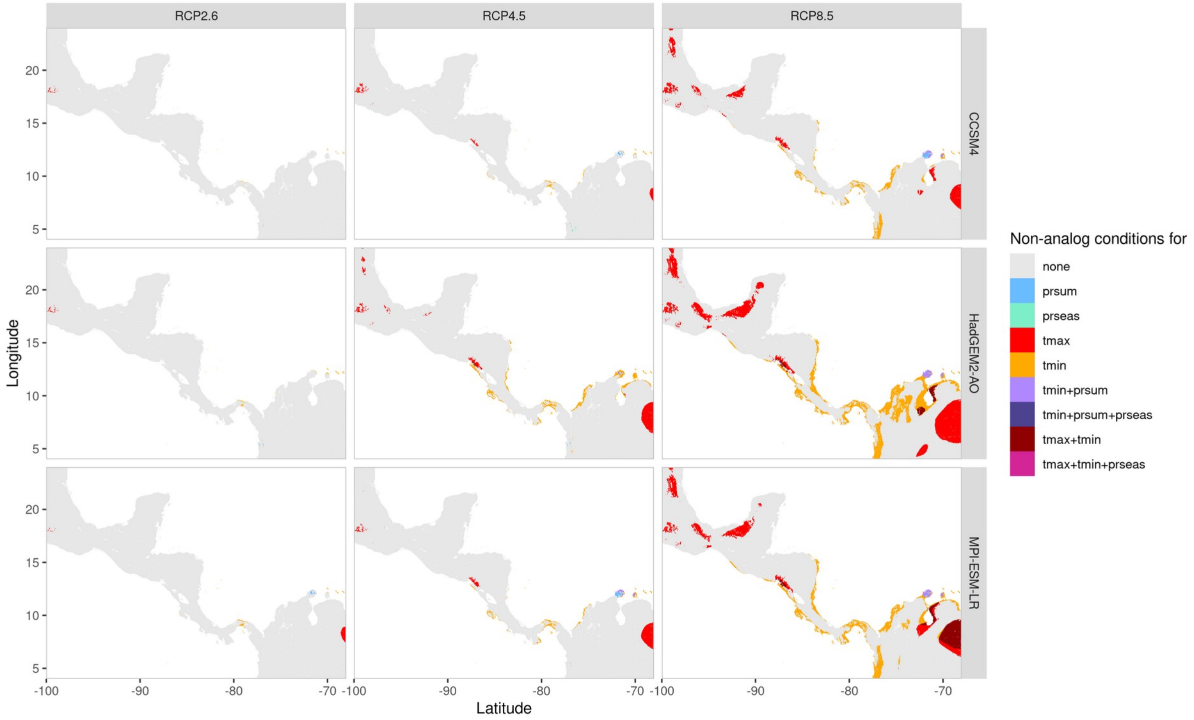

Figure S11: Non-analog conditions for annual precipitation (prsum), precipitation seasonality (prseas), maximum temperature (maxtemp) and minimum temperature (mintemp) compared to present-day climate data.

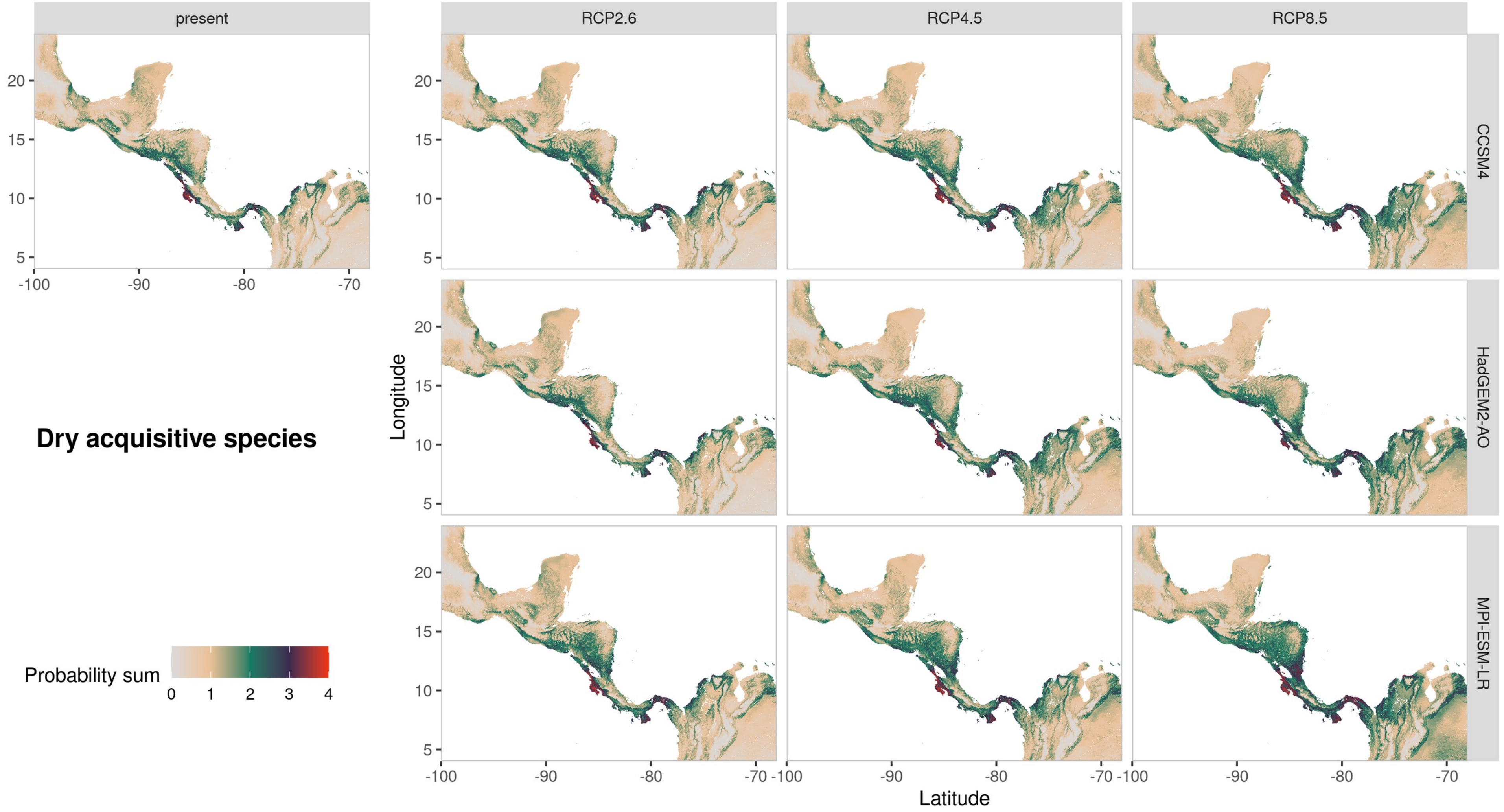

Figure S12: Sum of occurrence probabilities for dry acquisitive species.

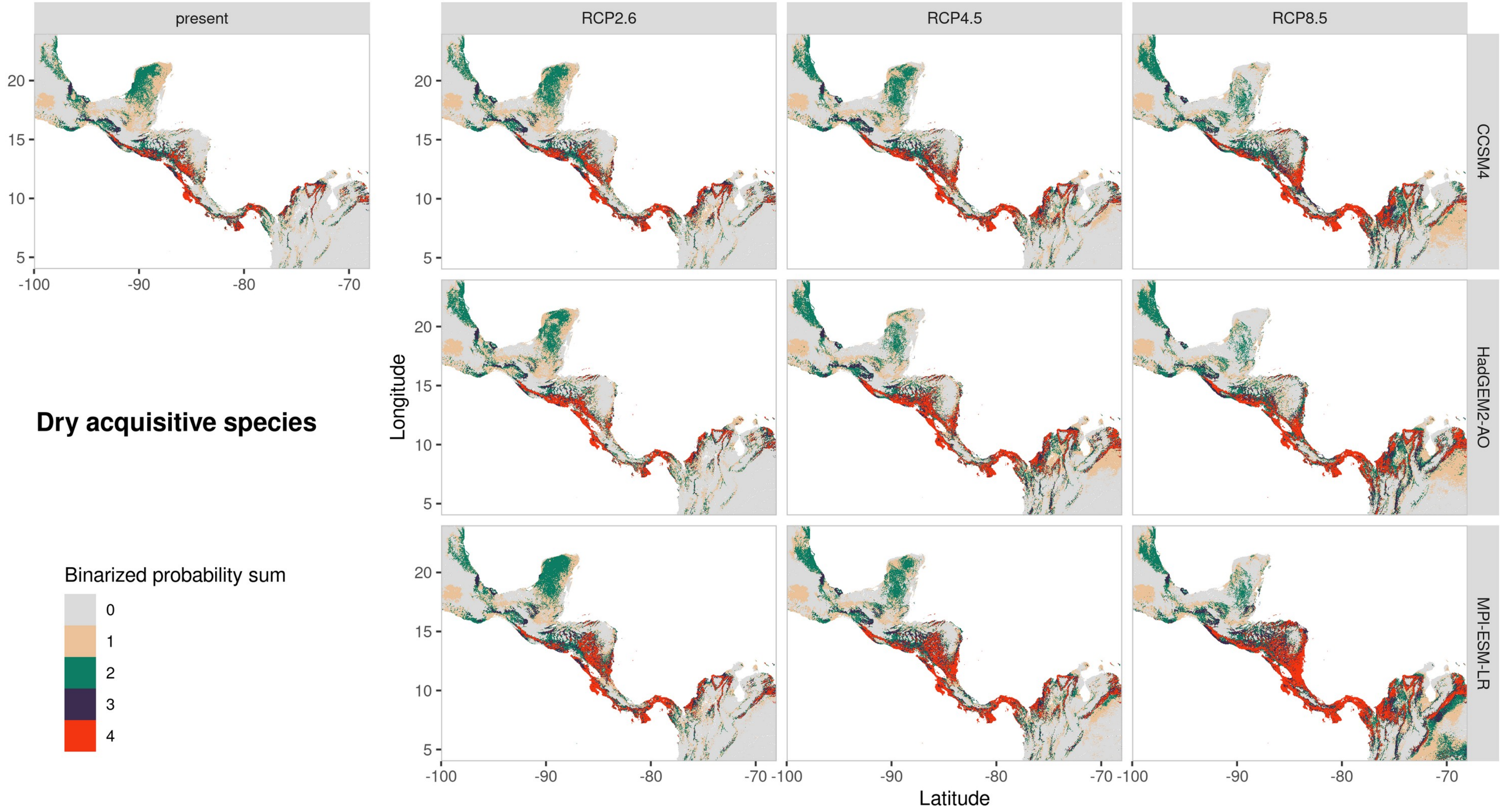

Figure S13: Sum of binarized projections for dry acquisitive species.

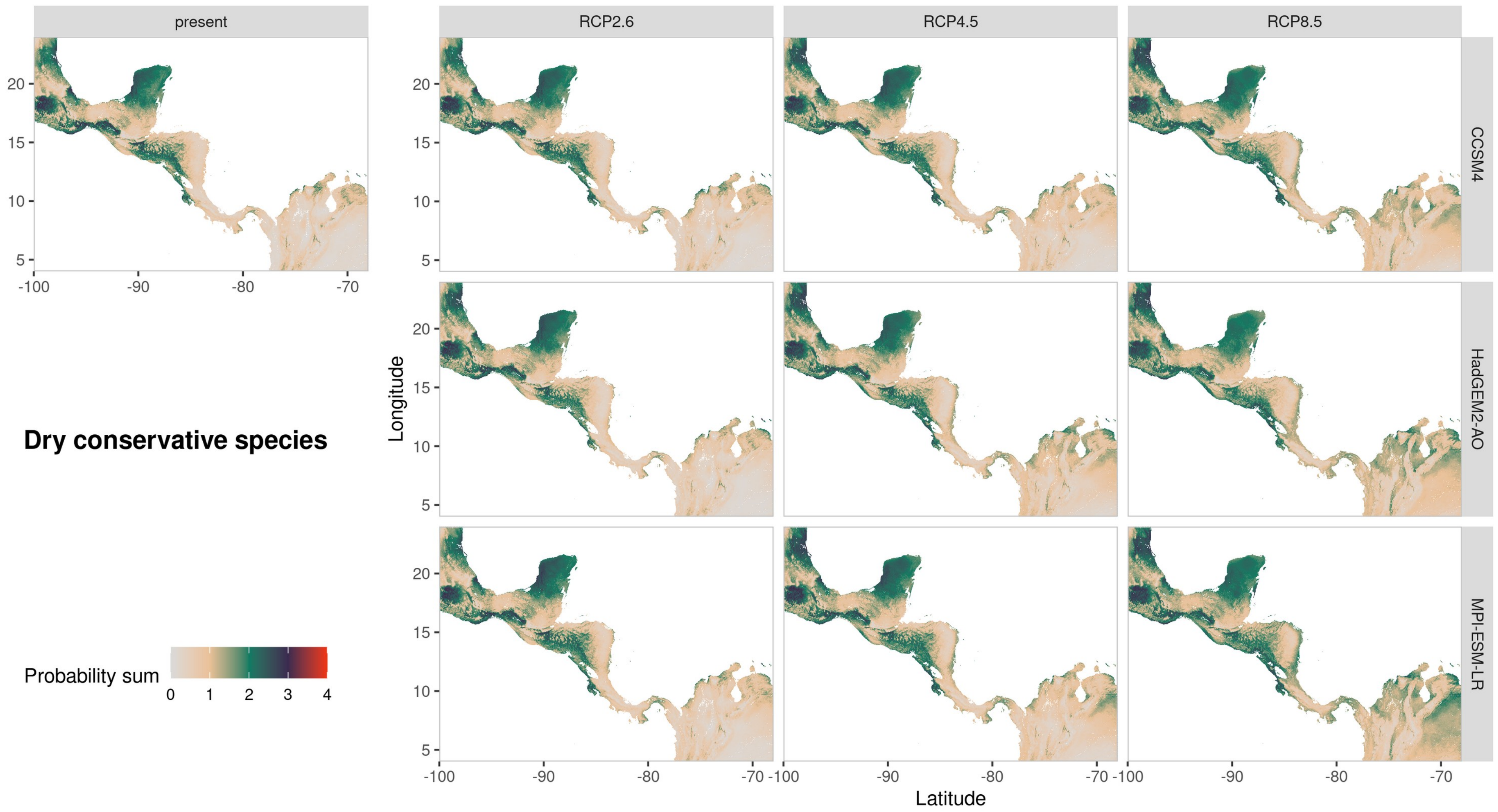

Figure S14: Sum of occurrence probabilities for dry conservative species.

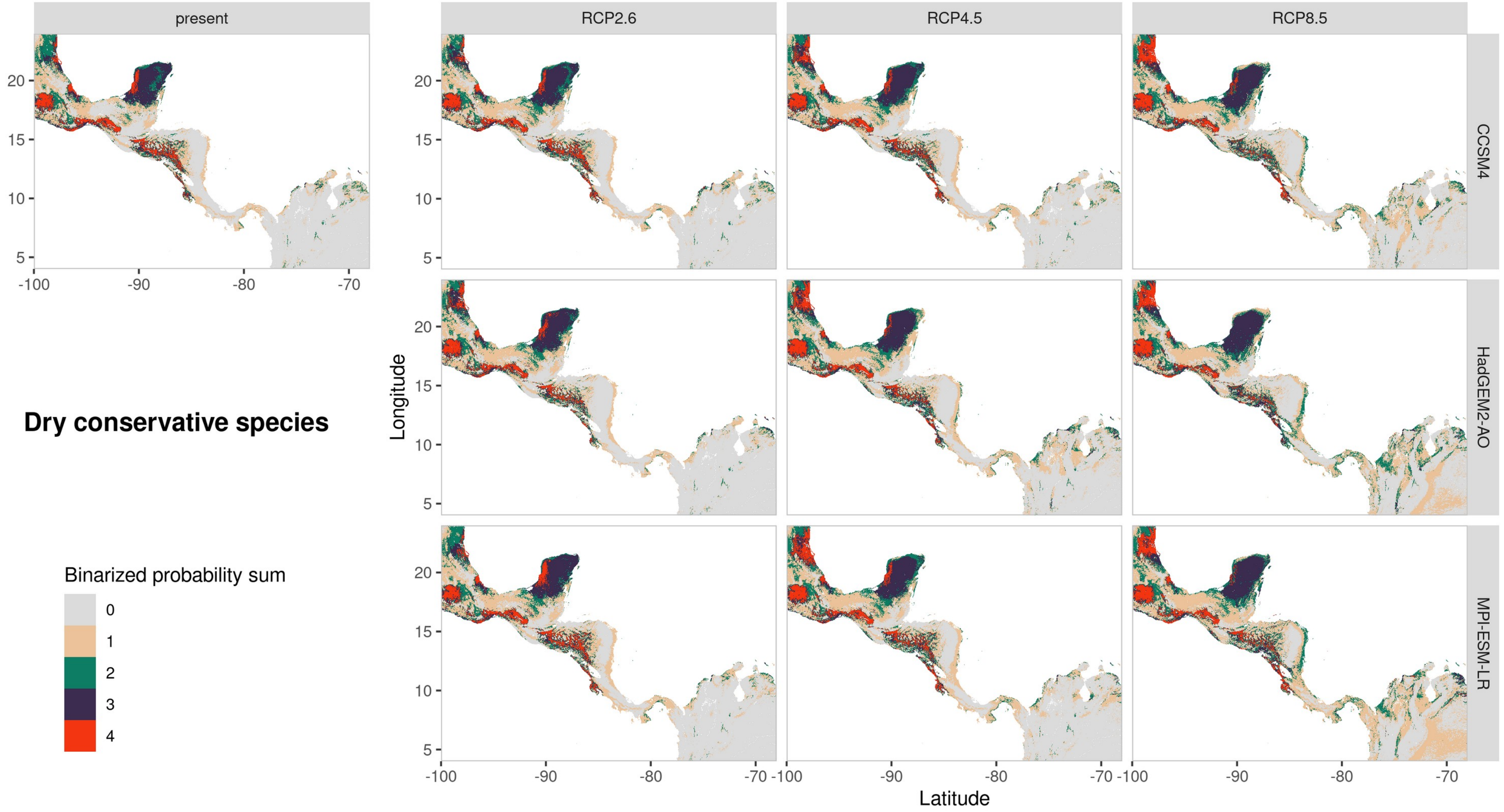

Figure S15: Sum of binarized occurrence probabilities for dry conservative species.

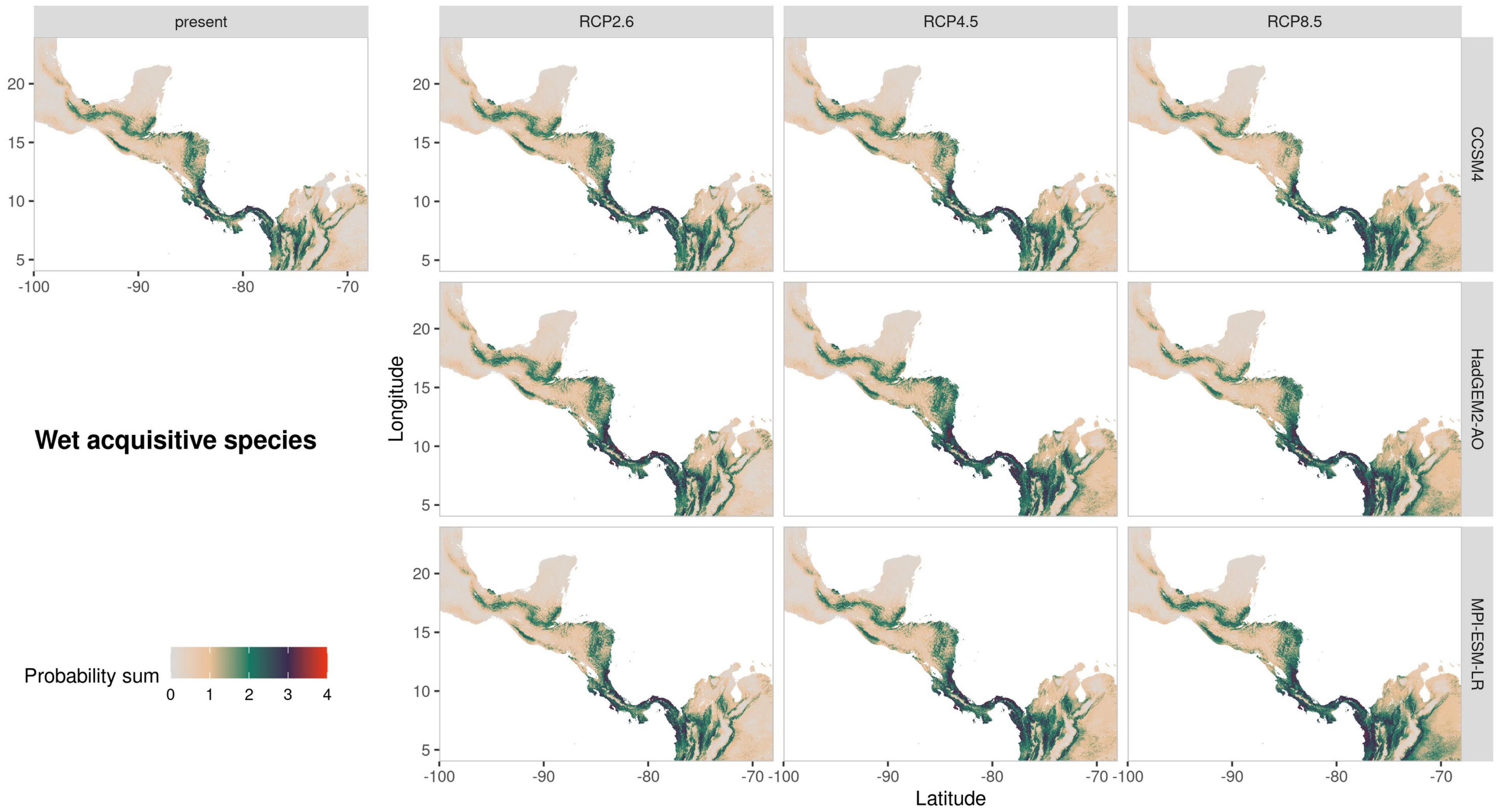

Figure S16: Sum of occurrence probabilities for wet acquisitive species.

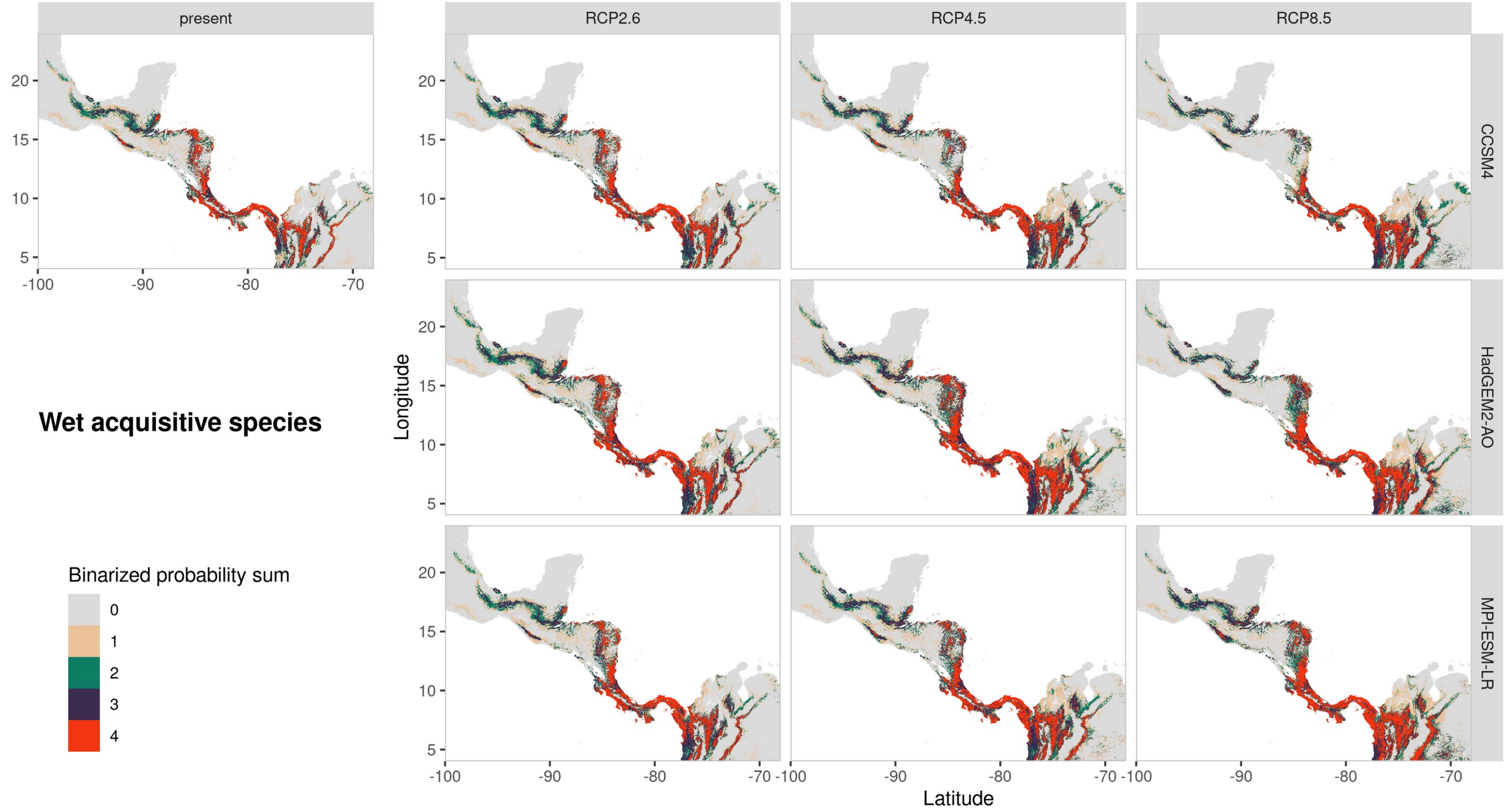

Figure S17: Sum of binarized occurrence probabilities for wet acquisitive species.

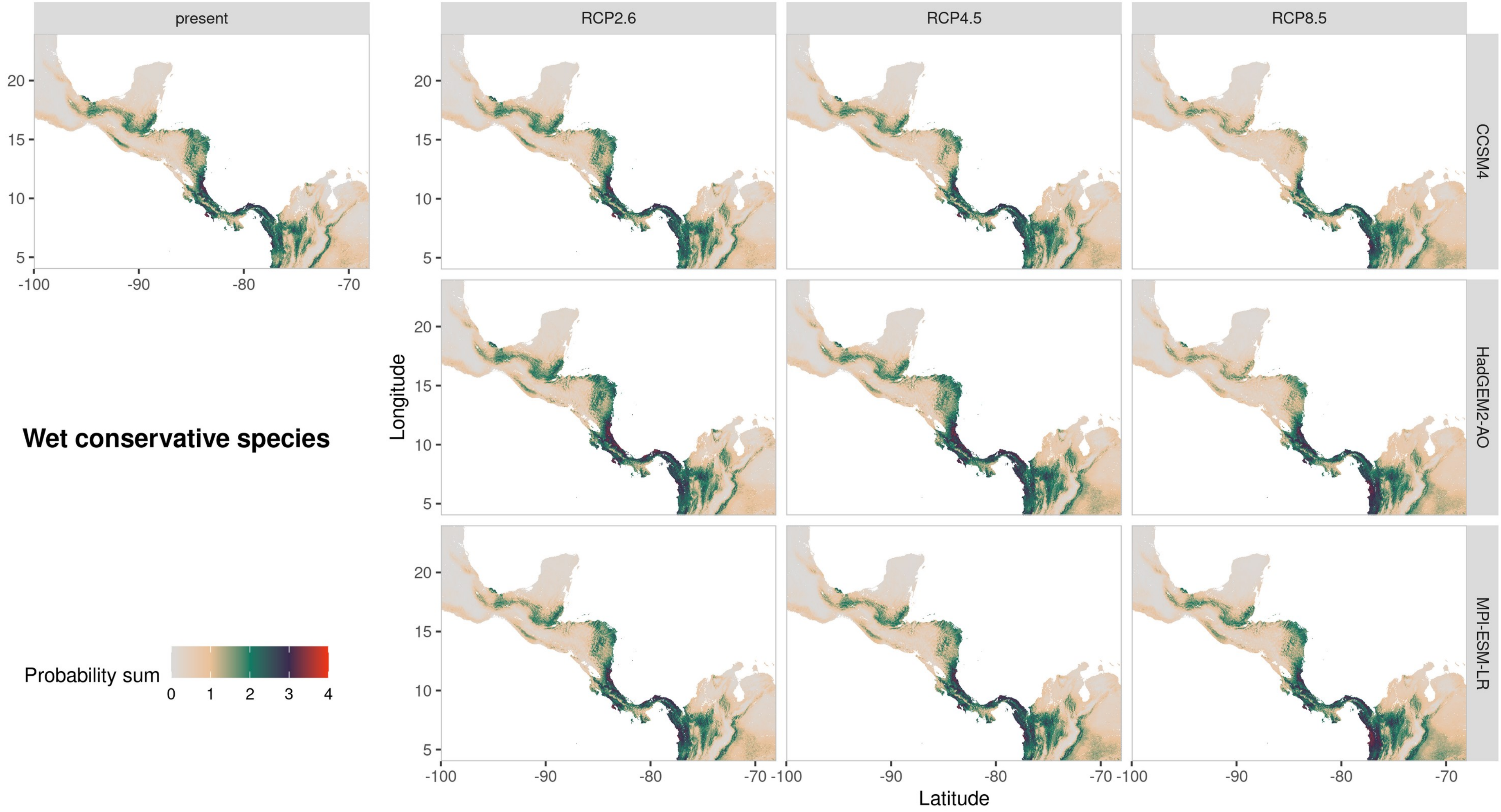

Figure S18: Sum of occurrence probabilities for wet conservative species.

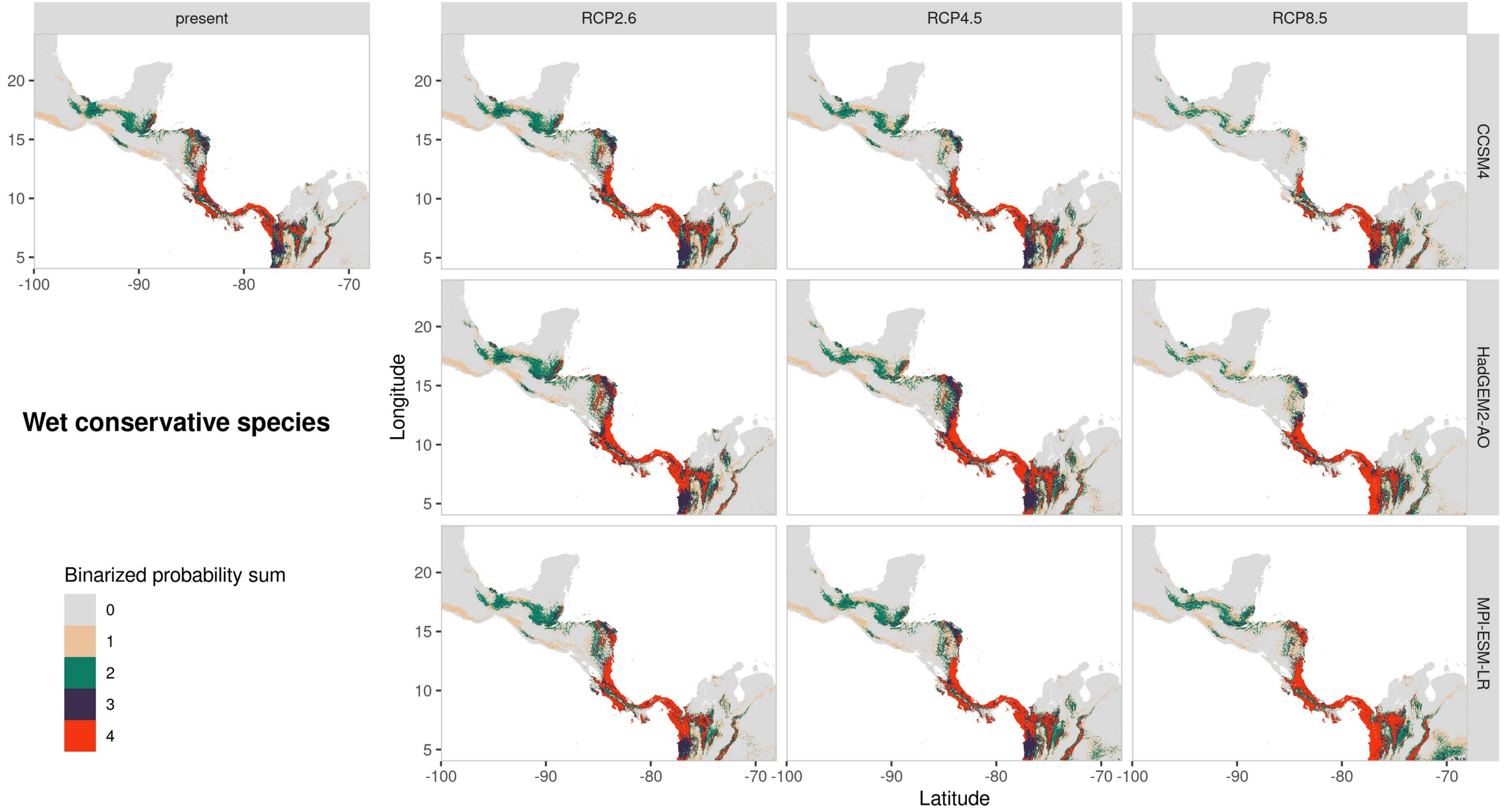

Figure S19: Sum of binarized occurrence probabilities for wet conservative species.

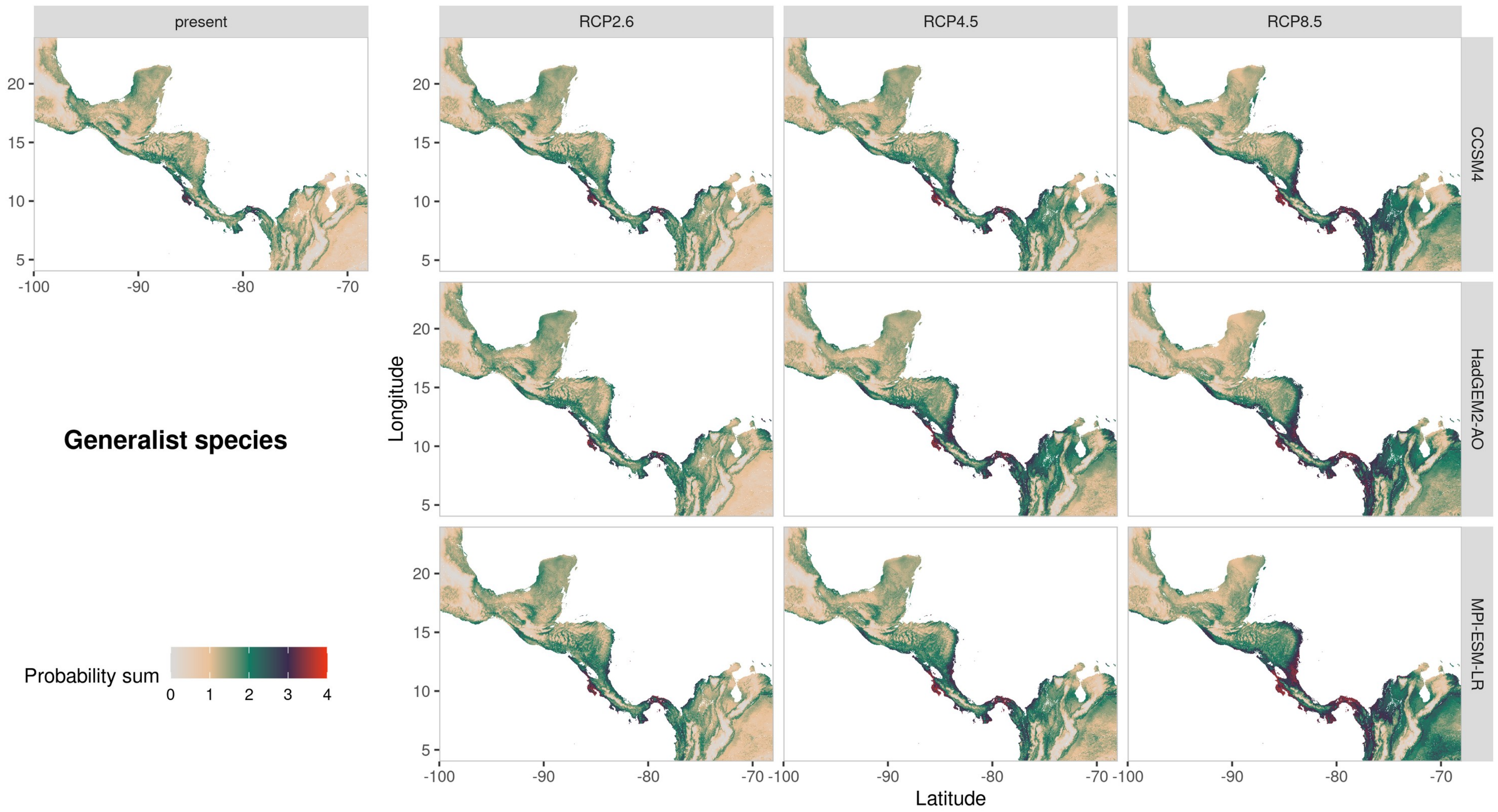

Figure S20: Sum of occurrence probabilities for generalist species.

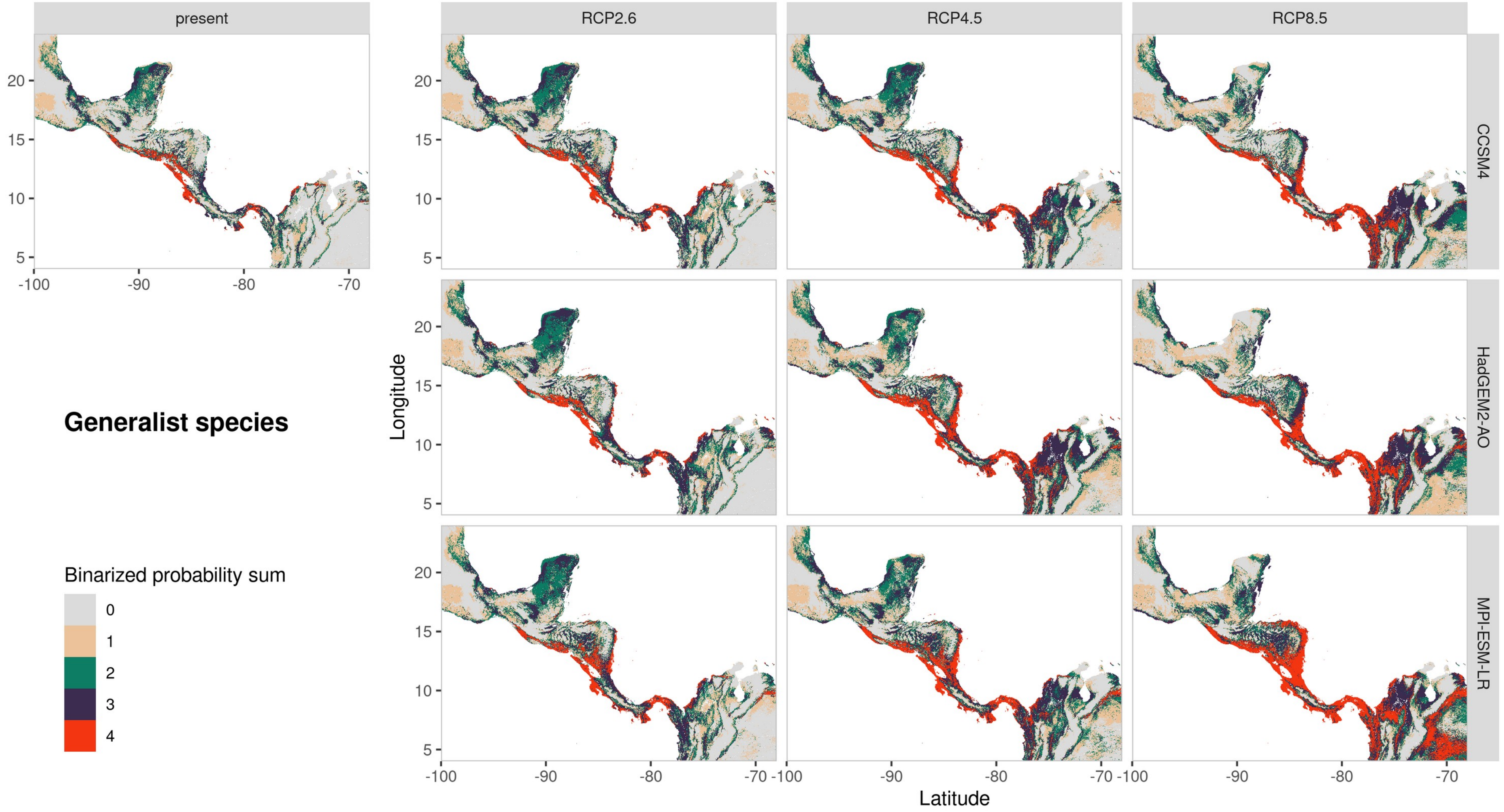

Figure S21: Sum of binarized occurrence ceprobabilities for generalist species.

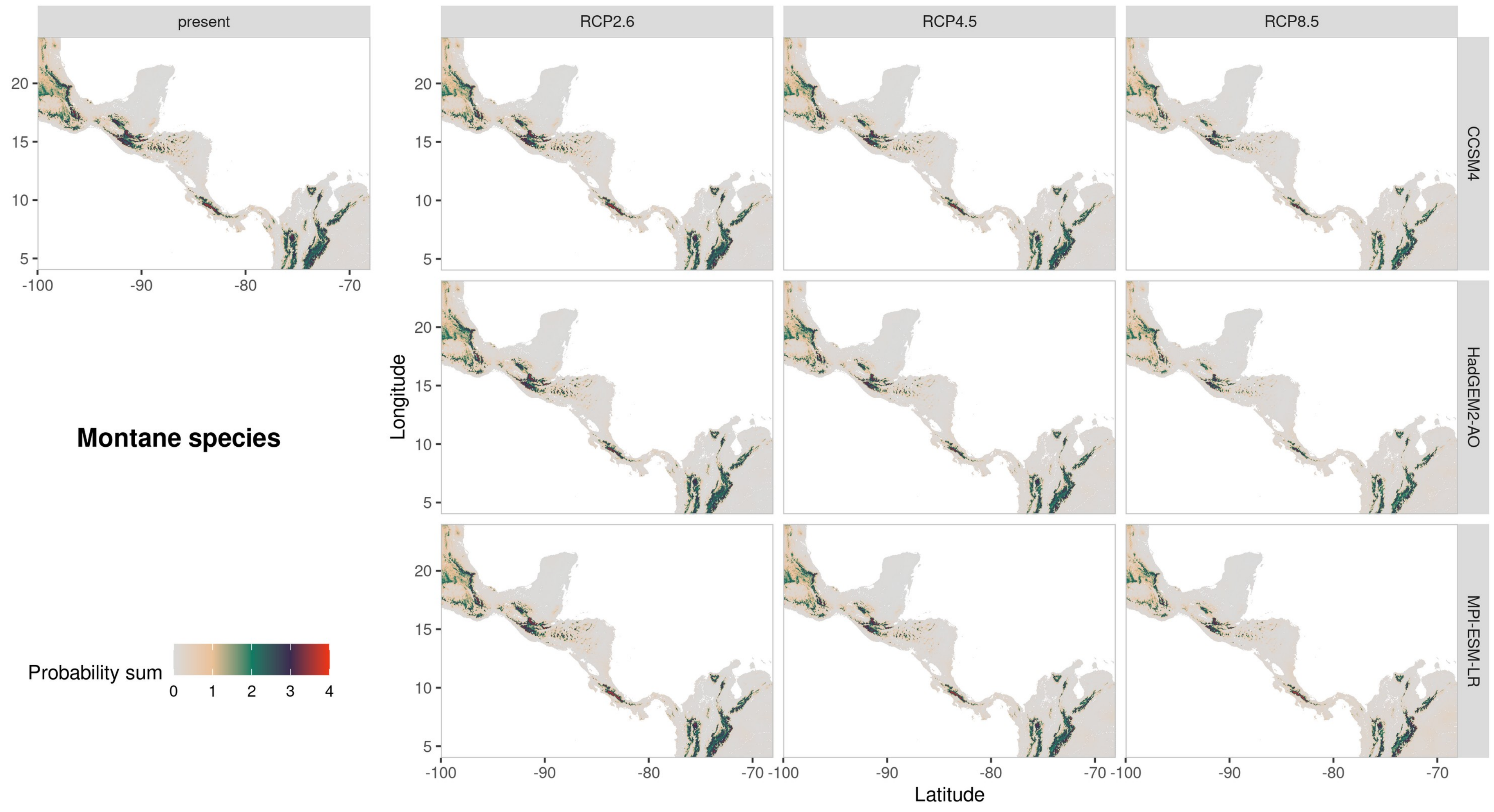

Figure S22: Sum of occurrence probabilities for montane species.

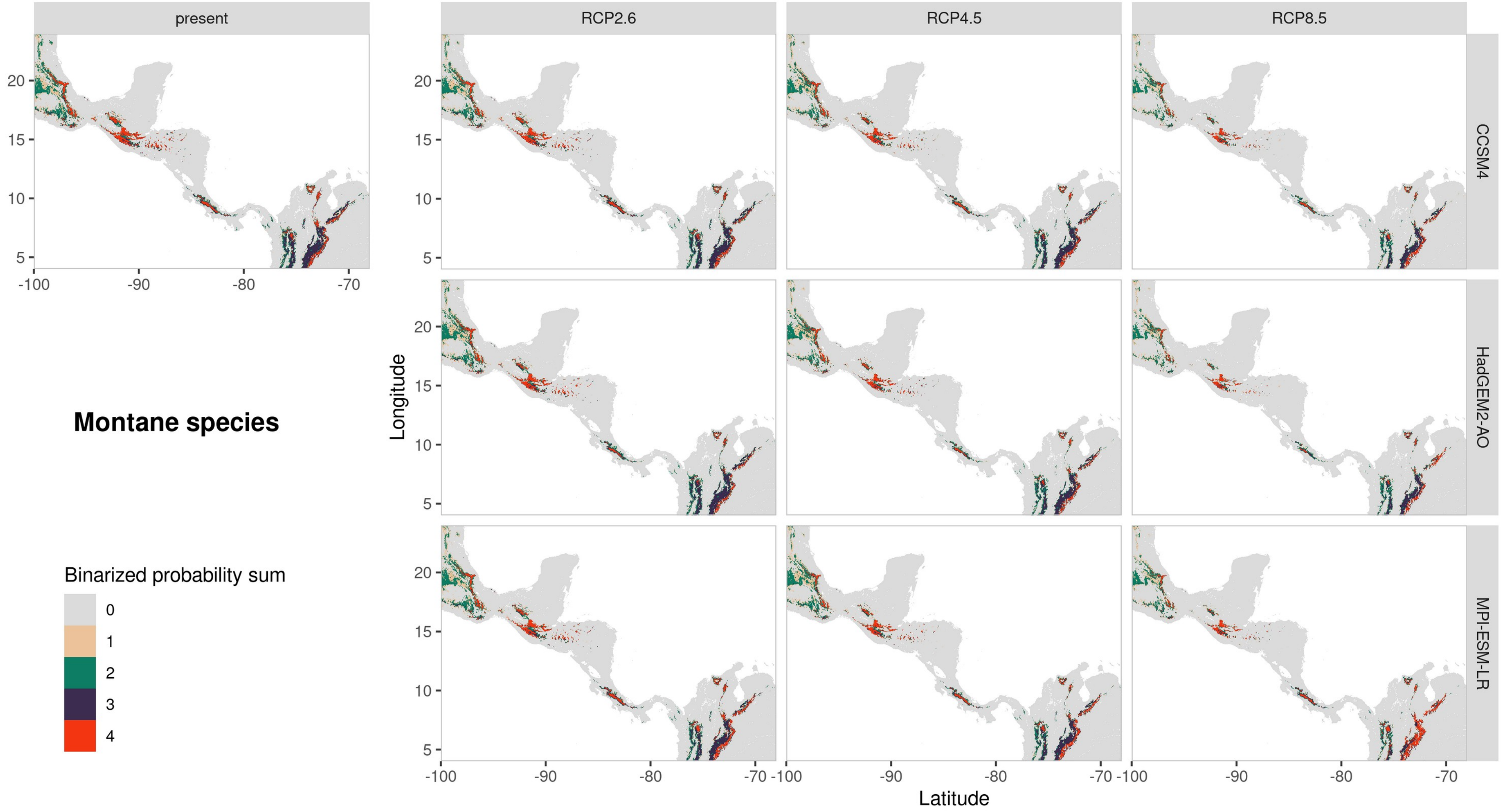

Figure S23: Sum of binarized occurrence probabilities for montane species.

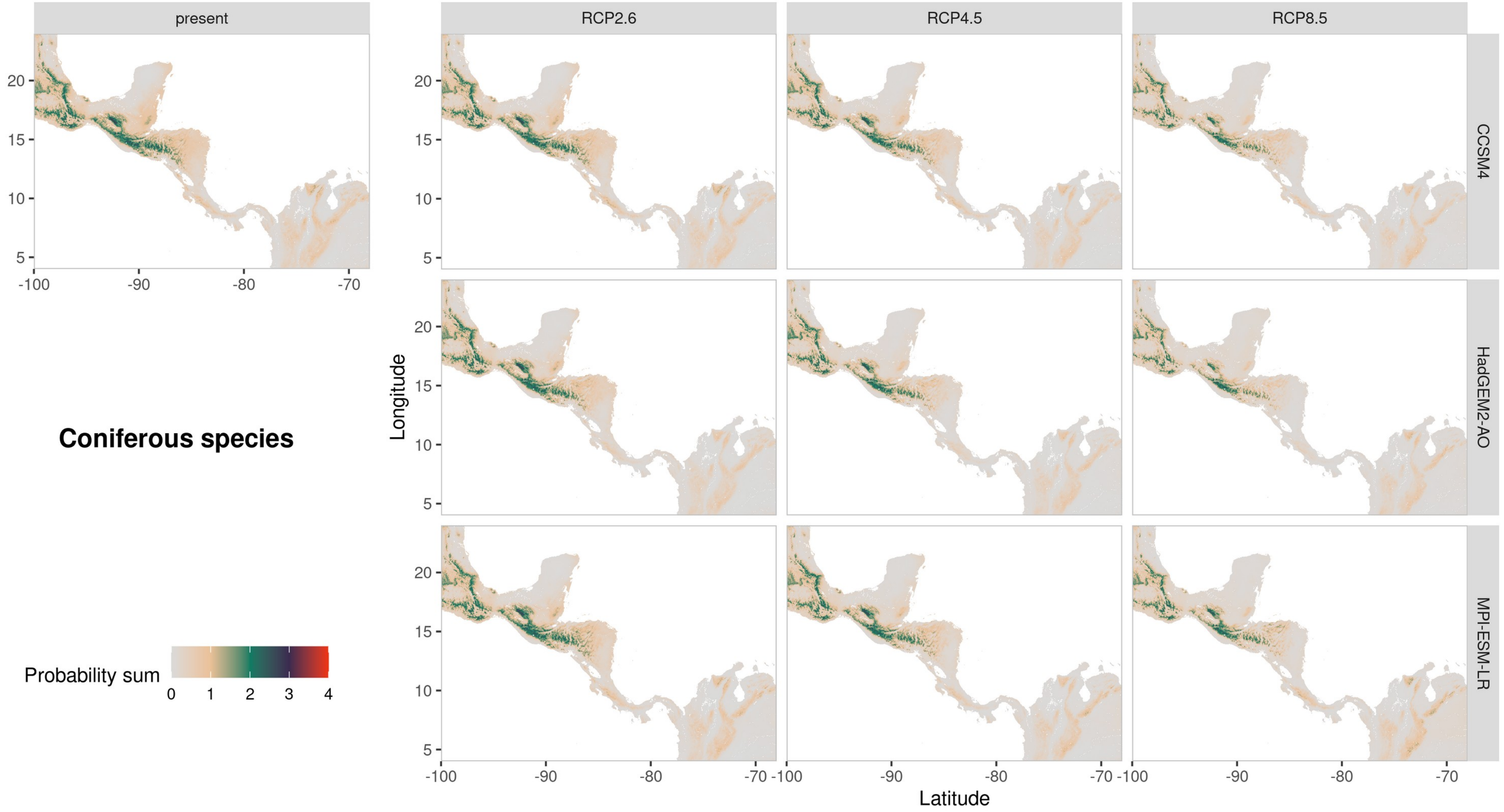

Figure S24: Sum of occurrence probabilities for coniferous species.

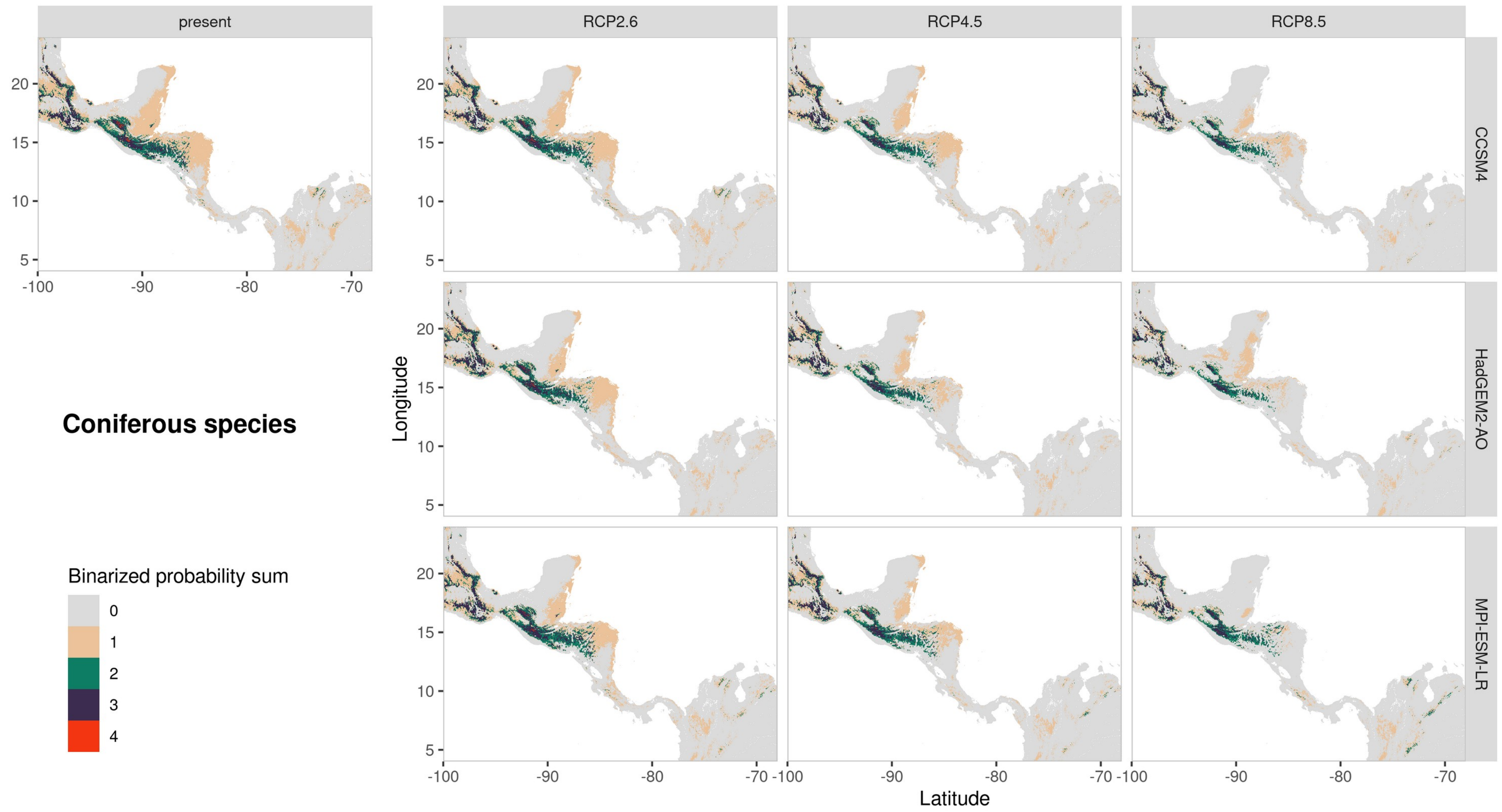

Figure S25: Sum of binarized occurrence probabilities for coniferous species.

Supplementary methods

PFT composition

For a general biogeographic division we largely followed the classification of terrestrial ecoregions by Olson et al. [1], which for Central America is based on the Holdrige Life Zone system [2]. Grounded in empirical studies, this system uses temperature, precipitation and altitudinal gradients to delineate biogeographical units. From these classifications, we adopted the main differentiation between tropical wet and dry forests. In Central America, this divide is marked by patterns of precipitation seasonality and roughly follows the Central American mountain ranges of the American Cordillera. Since the higher parts of these mountain ranges can experience freezing temperatures and thus accommodate distinctly adapted species, we additionally distinguish between lowland/premontane and montane forests (for a topographic overview see Figure S26).

Beyond these large-scale divisions, regional and local ecosystem patterns depend on a large number of factors including hydrological properties (e.g. runoff, water-logging), soil properties (e.g. pH, texture, nutrient availability) and light availability. As a result, tree species pursue specialized resource acquisition strategies to maximize their fitness [3]. For simplicity, here we cover this functional component by making a general differentiation between acquisitive and conservative species [4,5]. Acquisitive species, as their name suggests, tend towards fast resource acquisition by primarily investing energy in rapid growth at the cost of low structural quality (low wood density, cheap leaves). They maximize assimilation with high specific leaf area, high leaf nitrogen and phosphorus contents, shed their leaves during droughts and follow r-selection strategies with many rather small seeds [6]. Conservative species on the other hand invest more energy into robust tissue (dense wood, tough leaves) at the expense of lower growth rates. They are commonly (semi-) evergreen, have lower specific leaf area and follow a k-selection strategy with fewer but large seeds. In tropical wet forests conservative species are typically shade-tolerant and late-successional, while in dry forests they are shade intolerant and may rather be found in early-successional stages under harsh environmental conditions [7]. Despite our broad categorisation, some species still escape clear assignment, occurring widespread throughout Central America in

both wet and dry biomes and showing intermediate traits between acquisitive and conservative. To account for these non-specialist species, we included an additional type termed ”generalists”. Finally, while most Central American tree species are broadleaved, pine-dominated coniferous forests extend across coastal and montane areas in Honduras and the highlands of Guatemala. Since they are unique in their history of fire-driven succession [8] and are of special economic importance [9], we added them as a separate type.

A preliminary list of eligible species was created mainly based upon the compendium of Central American tree species by Cordero & Boshier [10], which already featured a grouping of species into Holdridge Life Zones. We thinned this list of species “candidates” by referring to information on these species in other guides (as cited in the main text), checking for occurrence data availability (>200) in the cited data bases and retrieving and checking trait data (for wet and dry acquisitive/conservative). To avoid including highly specialized or region-specific species, we further filtered the list by widespread abundance and large overlap of species presence records with the forest types they were suppposed to represent. To validate the latter two points we compared the distribution of occurrence data for each PFT with ecoregions [1], which is shown in Figure S27. Final selections were discussed and refined with the help of regional plant experts (Dr. J. Franciso Morales, Dr. Lenin Corrales and Dr. Bryan Finegan), which for example led to the decision of grouping Weinmannia species together due to close co-occurrence and easy confusion in the field.

Model setup

The amount of pseudo-absences and the method used for their selection are known to be a major factor of uncertainty in SDMs and influence their performance. What is more, depending on the model structure, different modelling methods show different sensitivity towards prevalence and spatial or environmental biases in the data. Barbet-Massin et al. [11] thus recommend to adapt the strategy used for pseudo-absence-sampling to the modelling algorithm used. Therefore, the model setup differed slightly between the algorithms used (see Table S2). In addition to the findings of Barbet-Massin et al. [11], during a test run we noted that for the classification and machine learning methods calibration increased with the number of pseudo-absences sampled - however at the cost of discrimination capacity. To

balance this trade-off, we moderately increased the number of pseudo-absences from their suggestion. For all presence-absence models we randomly sampled pseudo-absences with spatial exclusion of a 3km buffer around the presence locations (spatially stratified sampling), selecting twice as many pseudo-absences as presences but at least 1000.

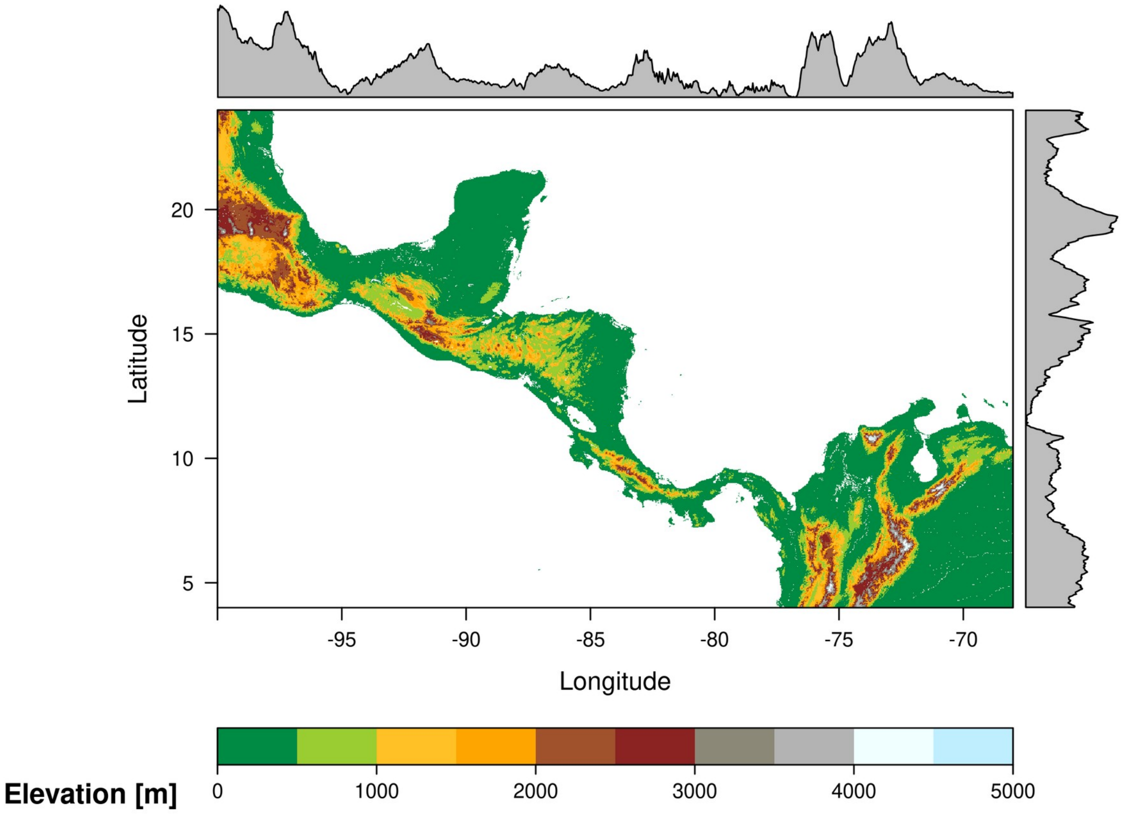

Figure S26: Elevation map of the study region (data: GMTED2010). Margin plots show mean elevation as a histogram along latitudes and longitudes, respectively.

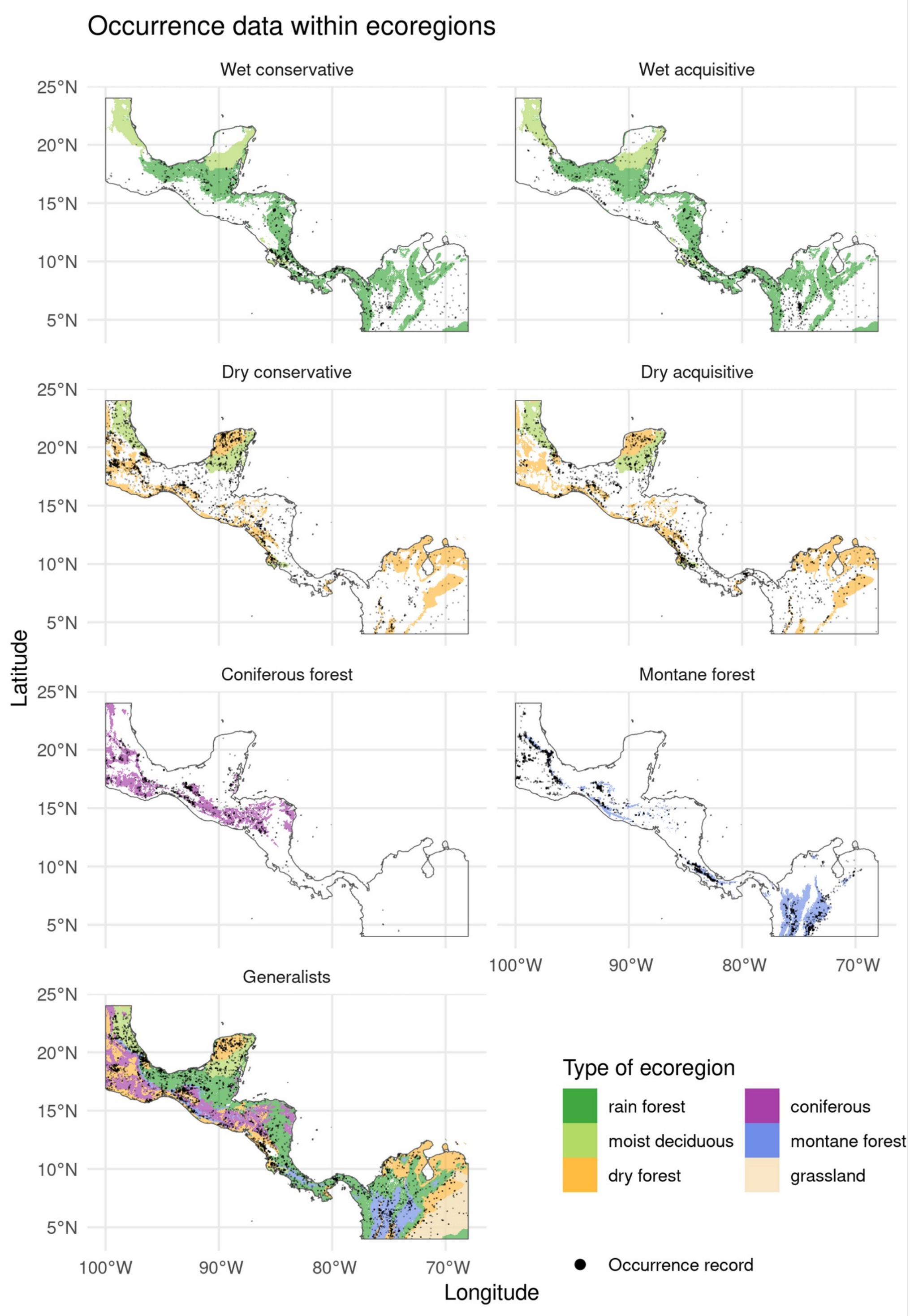

Figure S27: Distribution of occurrence data within ecoregions.

For MAXENT we randomly sampled 10’000 points throughout the study region to be used as background data. Since the step of pseudo-absence generation introduces additional uncertainties, we followed a Monte-Carlo approach by running 100 model replicates per

algorithm and species. Finally, because non-standardized predictors may skew responses of ANN and SVM (giving unequal weight to the variables), we applied min-max normalization to the environmental data in these runs (for the climatic variables throughout historic and scenario data). For a full summary of our settings and study design, see the protocol of the newly developed standard ODMAP [12] (section ODMAP protocol).

Table S2: Model setup.

algorithm(s)	sampling strategy	number of absences	data normalization
GAM, GLM, MARS	spatially stratified	presences × 2, min. 1000	no
CTA, RF	spatially stratified	presences × 2, min. 1000	no
ANN, SVM	spatially stratified	presences × 2, min. 1000	yes
MAXENT	random	10’000 background points	no

Cascade of uncertainties

Occurrence data uncertainty. Despite putting great care in the selection of occurrence points for model training, errors in the collection of species records can not be precluded. Due to common physiological features and high species diversity in tropical forests, taxonomic misidentifications in the field can still be a relevant issue [13]. Yet, since in this study we mostly work at the level of trait-based species groups we do not expect these errors to be critical. Other data issues may stem from sampling bias and precision of coordinates. The spatial thinning of occurrences is expected to reduce these impacts, yet edge effects between grid cells likely persist. The lack of true absence data represents another common problem for SDMs. Here, we followed a Monte-Carlo approach to account for the effect of pseudo-absence sampling. The ANOVA results showed, however, that this is a minor source of variance. Finally, we also consciously chose a low number of species per type to avoid an inflation of model uncertainties in the process of SDM stacking.

Predictor variable uncertainty. The choice of predictor variables is known to constitute a critical factor in the model building process [14]. Over the years, different strategies for the selection of environmental predictors for SDMs have evolved, which may mainly be divided in a-priori and a-posteriori methods. The former embrace fully exploratory approaches which

include a large set of potentially important variables, variable aggregation using principal component analysis or restricted selection based on ecological understanding [14,15]. A-posteriori methods on the other hand may consist in a backwards selection of environmental predictors based on their relative contribution to explained variance [16]. To reduce model complexity and avoid overfitting, in this study we decided to include only a few but biologically meaningful predictors [17,18]. Since the importance of predictor variables may vary between species, a unique predictor set for each group could probably improve accuracy, but would also impede comparison across models. Furthermore, inaccuracies in the predictor variables add to the list of uncertainties. Since most environmental data themselves are products of models (often run at a global scale), uncertainties inevitably propagate throughout the modelling process of SDMs. An evaluation of climate projections for Central America showed that these generally performed well for mean temperature but could not precisely reproduce precipitation and monthly temperature patterns and showed low skill for estimating the El Nino Southern Oscillation effect [19]. By using a range of less closely related well-ranking GCMs [20] we aimed to provide a spectrum of projections and analysed their standard deviation to highlight centres of uncertainty. More recently, the effect of spatial autocorrelation (particularly of residuals) in SDMs has been brought to new attention [21]. While there is broad agreement that the dependence of closeby predictor data violates a fundamental principle of classical statistics, the effects on model results are not clearly understood yet. One of the common tenors is to use a Bayesian modelling framework instead of frequentist methods, however, this was beyond the scope of our study.

Algorithm uncertainty. One of the major sources of uncertainty lies in the choice of modelling algorithm used to build SDMs [22,23]. Due to fundamental differences in the model fitting process, species responses in environmental space may vary greatly between the methods [24]. Here we used model ensembling to counteract these differences. While there is general agreement in the literature for using ensembles opposed to one single method, still it should be noted that a “best model” may be overruled by this strategy.

Analysis of Variance. To uncover main uncertainty sources, we conducted an analysis of variance in dependance of model replicates, modelling algorithms, RCPs and GCMs for each species (Fig. S28). The resulting total sum of squares was highest for modelling algorithms,

highlighting a high impact of algorithm choice on predictions. Model replicates (i.e. varying subsets of occurrence data used for model training) had the lowest impact on variance. Nevertheless, additional important sources of uncertainty might have been missed, since the unexplained variance (residuals) remained high for many species.

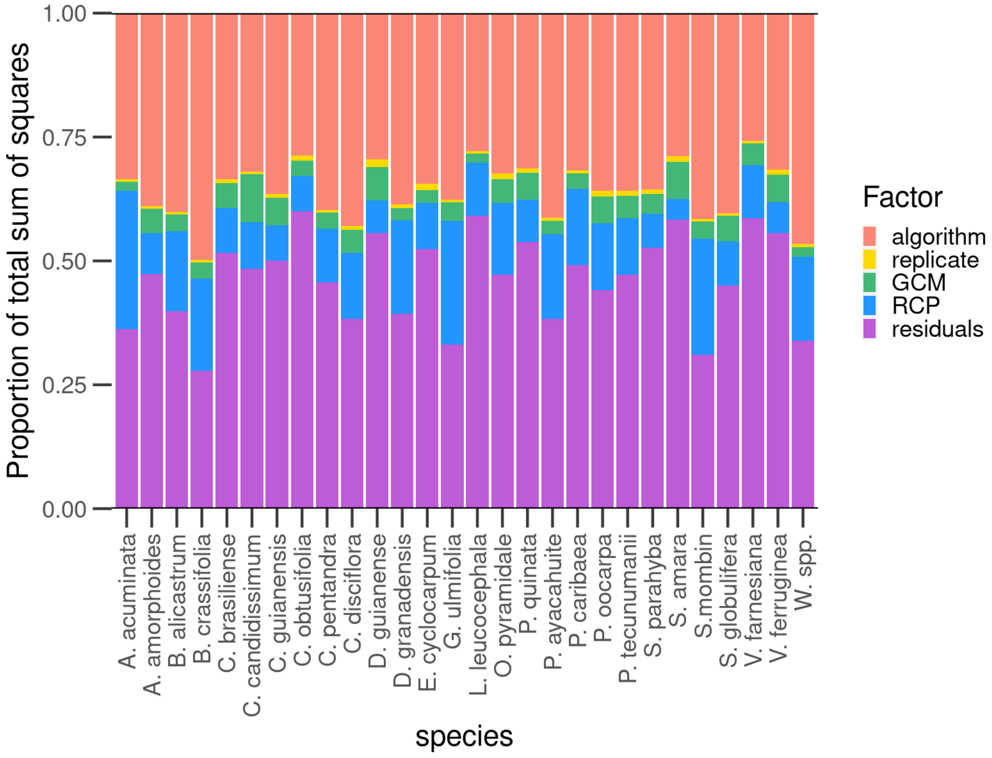

Figure S28: Average proportion of the ANOVA sum of squares of different sources of uncertainty (choice of algorithm, model replicate (training data sample), global circulation model (GCM), representative concentration pathway (RCP) and residual error).

Uncertainty maps. To investigate spatial patterns of uncertainty, we computed the variance (V ar) and standard deviation of the stacked projections (S) from the sum of predicted occurence probabilities (p) of each model ensemble within a PFT multiplied by the inverse probability (1 − p) [25]:

$$Var(S) = \Sigma(1 - p)\, p$$

The standard deviation was then calculated as square root of the variance (Fig. S29-S31).

For most PFTs and scenarios, the highest uncertainties occurred in the areas with the highest suitabilities for these types. Particularly the generalist type showed high deviations across most of the study region. This result is mainly related to the parabolic relationship between occurrence probabilities and variance as seen in the above equation. Consequently, occurrence probabilities close to the range ends result in very low variance, while variance increases strongly towards the centre of the distribution. Since our models were not optimized for high discrimination (i.e. strong separation between presences (1) and absences (0)), many predictions were in the range of 0.2-0.8 and thus lead to higher variances. Since all types were affected similarly by this effect, the impact on our study results are expected to be minor.

Recalibration

The recalibration of original suitability scores predicted by SDMs offers an opportunity to improve model calibration and allow for probabilistic interpretation of the output. Since the implementation can be a computationally expensive process, we tested a subset of five species ensemble models (GAM, MARS, MAXENT and RF for A. amorphoides, B. alicastrum, B. crassifolia, C. brasiliense, C. calycophyllum). We applied three state-of-the-art calibration methods and subvariants using the CalibratR package [26] and evaluated their success with expected calibration error (ECE), mean calibration error (MCE) and Continuous Boyce Index (CBI). The evaluation showed that all calibration methods (except raw transformed) reduced calibration error, however only to a small degree (Table S3). CBI only improved for the histogram transformed method, but reduced for all other methods, particularly GUESS. The resulting recalibrated maps presented suitability patterns that strongly differed from the original predictions and were ecologically questionable: ranging from predicting only a few pixels as presences to drawing complete negative images compared to the original predictions. Based on these experiences, we found the original predictions to be sufficiently well calibrated and continued with these.

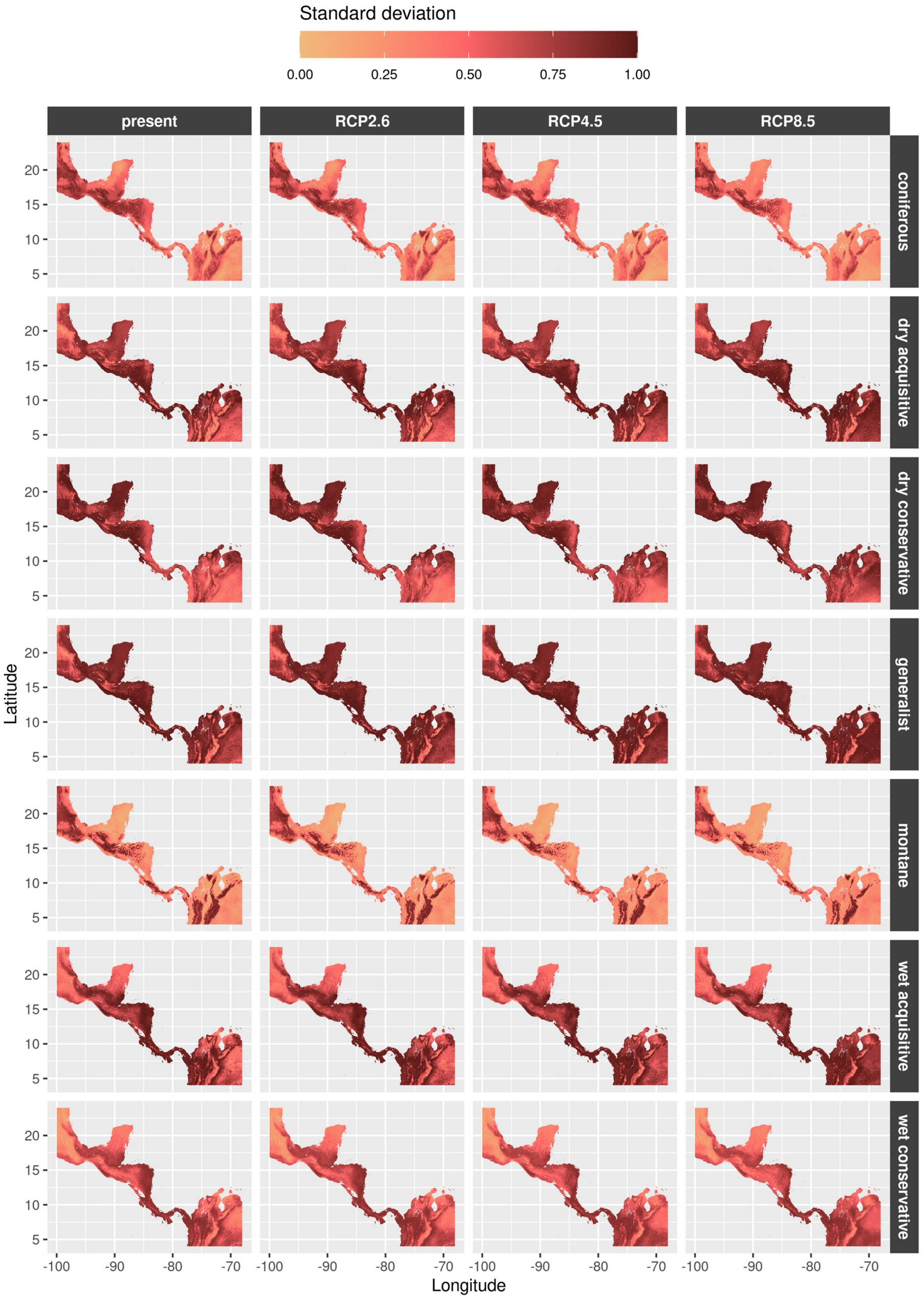

Figure S29: Standard deviations of the stacked projections for the CCSM4 scenarios.

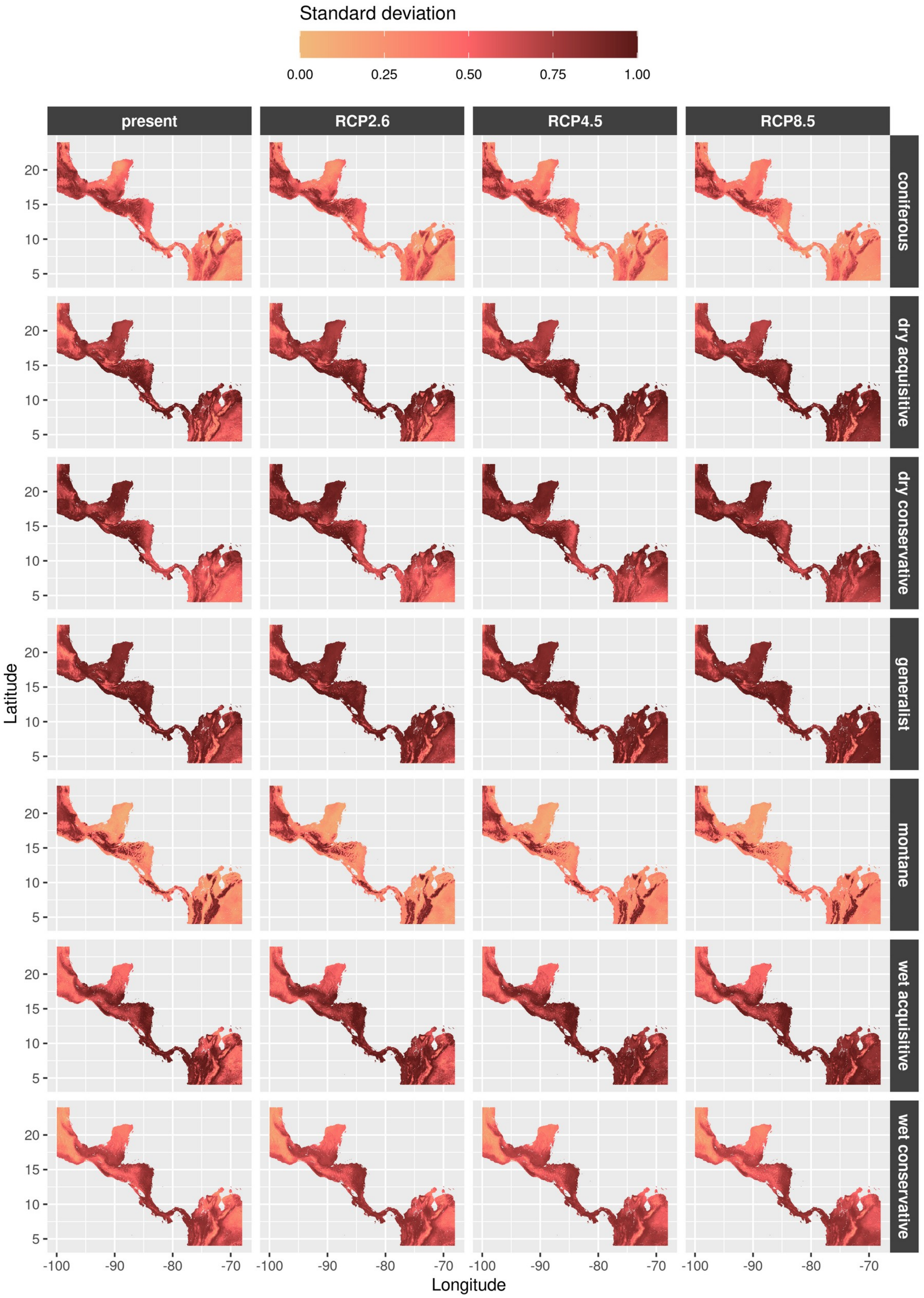

Figure S30: Standard deviations of the stacked projections for the HadGEM-AO scenarios.

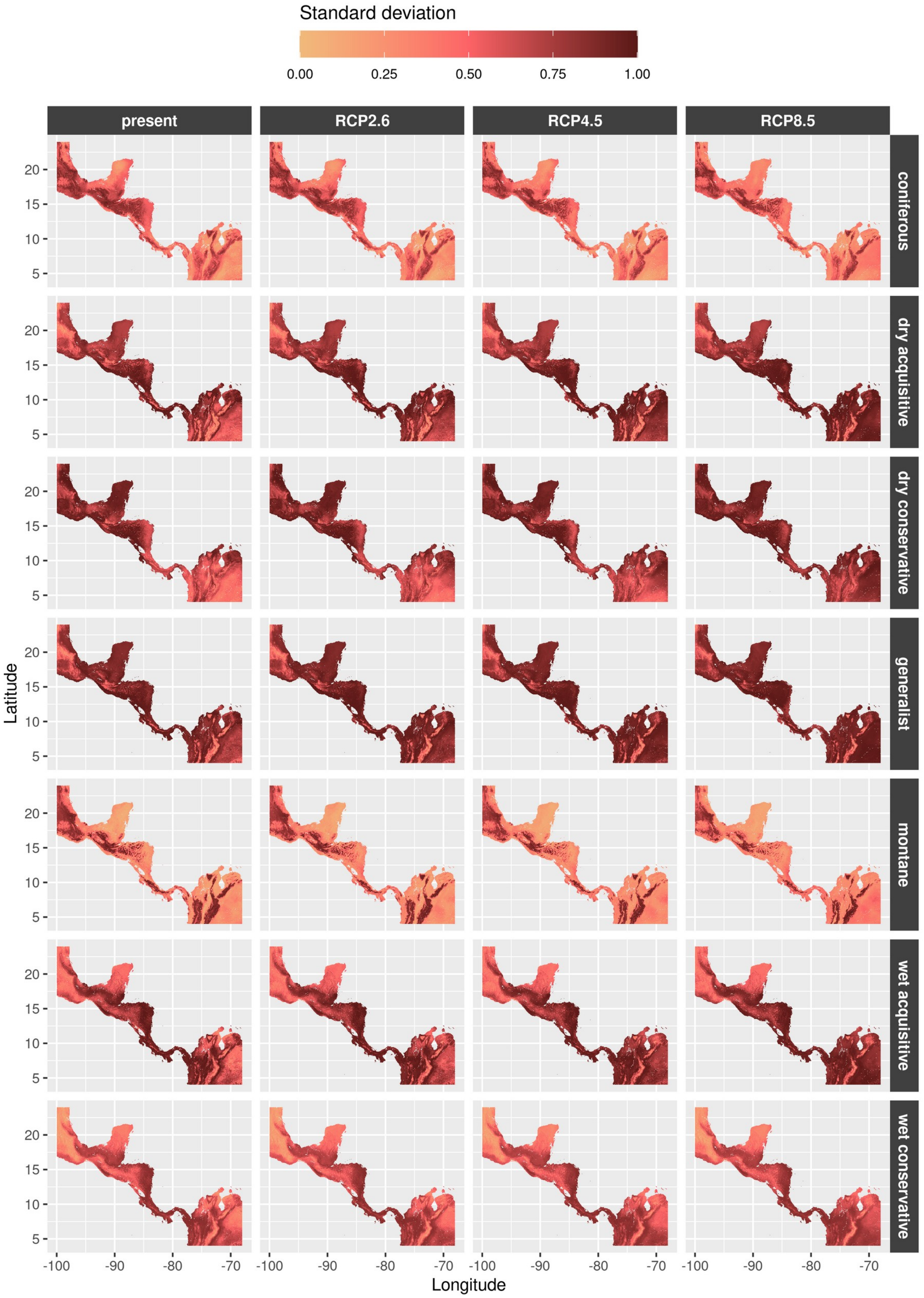

Figure S31: Standard deviations of the stacked projections for the MPI-ESM-LR scenarios.

Table S3: Recalibration statistics. Expected calibration error (ECE), mean calibration error (MCE), ECE/MCE with equal width bins (eq) and Continuous Boyce Index (CBI) statistics for calibration models of five species (all algorithms). Recalibration methods refer to those implemented in the CalibratR package [26].

method	ECE	MCE	ECEeq	MCEeq	CBI
original	0.217	0.047	0.204	0.054	0.971
scaled	0.217	0.047	0.204	0.054	0.858
transformed	0.304	0.043	0.297	0.081	0.969
Histogram scaled	0.036	0.009	0.015	0.005	0.783
Histogram transformed	0.026	0.007	0.004	0.003	1.000
BBQ scaled selected	0.033	0.008	0.018	0.006	0.871
BBQ scaled average	0.028	0.007	0.018	0.006	0.782
BBQ transformed selected	0.033	0.008	0.018	0.006	0.890
BBQ transformed average	0.028	0.007	0.018	0.006	0.803
GUESS 1	0.081	0.020	0.084	0.023	0.695
GUESS 2	0.040	0.012	0.039	0.013	0.635

ODMAP Protocol

This protocol was created online with the web application ODMAP v.1.0 (https://odmap.wsl.ch/). For the original description of the protocol see Zurell et al. [12].

Climate change may induce connectivity loss and mountaintop extinction in Central American forests

– ODMAP Protocol –

Lukas Baumbach, Dan L. Warren, Rasoul Yousefpour, Marc Hanewinkel

2020-06-24

Overview

Authorship

Contact : lukas.baumbach@ife.uni-freiburg.de

<Study link>

Model objective

Model objective: Forecast and transfer

Target output: suitable vs. unsuitable habitat, comparison across species

Focal Taxon

Focal Taxon: Groups of tree species with common resource acquisition traits from typical Central American forest biomes

Location

Location: Central America

Scale of Analysis
Spatial extent: -100, -68, 4, 24 (xmin, xmax, ymin, ymax)

Spatial resolution: 30 arc-second (~1 km)

Temporal extent: 1979-2013 (present climate) to 2061-2080

Temporal resolution: 70 years

Boundary: rectangle

Biodiversity data
Observation type: citizen science, field survey

Response data type: point occurrence, presence-only

Predictors
Predictor types: climatic, edaphic, topographic

Hypotheses
Hypotheses: Increasing temperatures and precipitation seasonality could favour dry forest species and lead to loss of suitable habitat for montane species

Assumptions
Model assumptions: Species occurrence is at equilibrium with environment

Algorithms
Modelling techniques: maxent, glm, mars, ann, randomForest, gam, cta, svm

Model complexity: no limitation

Model averaging: model ensembling by unweighted mean

Workflow
Model workflow: for each species and algorithm 10 model replicates were fitted using random training data subsets (70% training, 30% test), cross-validation with holdout data, model evaluation with AUC, calibration statistic and Continuous Boyce Index, model selection based on evaluation threshold (AUC/calibration/CBI >0.7), recalibration, model ensembling across algorithms and species (SSDM)

Software
Software: R 3.6.3, packages: SSDM, ENMTools, CalibratR

<Code availability>

<Data availability>

Data

Biodiversity data
Taxon names: Alnus acuminata, Alvaradoa amorphoides, Brosimum alicastrum, Byrsonima crassifolia, Calophyllum brasiliense, Calycophyllum candidissimum, Ceiba pentandra, Cornus disciflora, Dialium guianense, Drimys granadensis, Enterolobium cyclocarpum, Guazuma ulmifolia, Leucaena leucocephala, Ochroma pyramidale, Pachira quinata, Pinus ayacahuite, Pinus caribaea, Pinus oocarpa, Pinus tecunumanii, Schizolobium parahyba, Simarouba amara, Spondias mombin, Symphonia globulifera, Vachellia farnesiana, Vochysia ferruginea, Weinmannia spp. (anisophylla, balbisana, burseraefolia, burserifolia, elliptica, fagaroides, horrida, karsteniana, multijuga, pinnata, pubescens, rollottii, vulcanicola, wercklei)

Taxonomic reference system: GBIF Backbone Taxonomy

Ecological level: species, communities

Data sources: GBIF, BIEN, de Sousa et al. (2017) (see main article)

Sampling design: random

Sample size: species dependent ~200 to ~5600 (number of pseudo-absences was scaled accordingly)

Clipping: study extent (68-100W, 4-24N), masking by land area

Scaling: spatial thinning to one record per grid cell of environmental rasters

Cleaning: removal of duplicates, union of synonym taxa to GBIF accepted name

Absence data: not available

Background data: random generation of pseudo-absence data with 3km spatial exclusion around presences. MaxEnt: 10’000 randomly sampled background points.

Errors and biases: taxonomic misidentification possible

Data partitioning
Training data: random, 70%

Validation data: none

Test data: 30% withheld from model fitting

Predictor variables

Predictor variables: maximum temperature of the warmest month, minimum temperature of the coldest month, precipitation seasonality, annual precipitation, soil sand fraction (30cm depth), soil clay fraction (30cm depth), soil pH in water, depth to bedrock and hillslope

Data sources: climate/topography: http://chelsa-climate.org/downloads soil data: https://soilgrids.org/ slope: derived

Spatial extent: -100, -68, 4, 24 (xmin, xmax, ymin, ymax)

Spatial resolution: 30 arc-second

Coordinate reference system: EPSG:4326 (WGS84)

Temporal extent: climate: 1979-2013, topography: 2010, soil: 2013

Temporal resolution: not applicable

Data processing: grid alignment

Errors and biases: soil data sampling bias (mostly Pacific coast)

Dimension reduction: multi-collinearity check via variance inflation factor calculation, stepwise supervised exclusion of critical variables until VIF<10

Transfer data

Data sources: climate scenarios (CMIP5): http://chelsa-climate.org/downloads

Spatial extent: -100, -68, 4, 24 (xmin, xmax, ymin, ymax)

Spatial resolution: 30 arc-second

Temporal extent: 2061-2080

Temporal resolution: not applicable

Models and scenarios: CCSM4, HadGEM2-AO, MPI-ESM-LR, RCPs 2.6, 4.5 and 8.5

Quantification of Novelty: clamping to value range of original predictors used for model training

Model

Variable pre-selection
Variable pre-selection: environmental limits of ecological importance drawn from literature and regional experts

Multicollinearity
Multicollinearity: variance inflation factor (VIF)

Model settings
maxent: featureSet (Auto features), regularizationMultiplierSet (1), convergenceThresholdSet (0.00001)

glm: family (gaussian), formula (presence ~ x1 + … + xn), weights (NULL), test (AIC), epsilon (1e-08), maxit (500)

mars: formula (presence ~ .), degree (2), penalty (3), nk (automatically set), thresh (0.001), pmethod (backward)

ann: formula (presence ~ .), size (6), decay (0), maxit (500), trace (FALSE)

randomForest: ntree (2500), mtry (sqrt(nvars)), maxnodes (NULL), try (N/A), do.classif (TRUE), nodesize (1)

gam: family (gaussian), formula (presence ~ x1 + … + xn), smoothTerms (+ s(x1) + … + s(xn)), weights (NULL), offset (NULL), method (GCV.Cp), select (FALSE), test (AIC), epsilon (1e-08), maxit (500)

cta: formula (presence ~ .), minbucket (1), xval (3)

svm: formula (presence ~ .), type (eps-regression), gamma (1/length(data) -1), kernel (radial), epsilon (1e-08), cross (3)

Model settings (extrapolation): clamping

Model estimates
<Coefficients>

Analysis and Correction of non-independence
<Spatial autocorrelation>

Threshold selection
Threshold selection: maximum sum of sensitivity+specificity

Assessment

Performance statistics
Performance on training data: AUC, calibration plot statistic (Naimi et al. 2016), Continous Boyce Index

<Performance on validation data>

Performance on test data: AUC, calibration plot statistic (Naimi et al. 2016), Continous Boyce Index

Plausibility check
<Response shapes>

Expert judgement: map display

Prediction

Prediction output
Prediction unit: probability

Post-processing: none

Uncertainty quantification
Algorithmic uncertainty: ANOVA, standard deviation

Input data uncertainty: ANOVA, standard deviation

Scenario uncertainty: ANOVA, standard deviation

Novel environments: novel climate maps

Supplementary references

1. Olson, D. M. *et al.* Terrestrial Ecoregions of the World: A New Map of Life on EarthA new global map of terrestrial ecoregions provides an innovative tool for conserving biodiversity. *BioScience* **51**, 933–938 (2001).
2. Holdridge, L. R. *Life zone ecology*. (Tropical Science Center, 1967).
3. Hulshof, C., Martínez-Yrízar, A., Burquez, A., Boyle, B. & Enquist, B. Plant Functional Trait Variation in Tropical Dry Forests: A Review and Synthesis. in *Tropical Dry Forests in the Americas* (ed. Quesada, M.) 129–140 (CRC Press, 2013). doi:10.1201/b15417-9.
4. Delgado, D. *et al. Análisis de la vulnerabilidad al cambio climático de bosques de montaña en Latinoamérica*. (Centro Agronómico Tropical de Investigación y Enseñanza (CATIE), 2016).
5. Lohbeck, M. *et al.* Functional Trait Strategies of Trees in Dry and Wet Tropical Forests Are Similar but Differ in Their Consequences for Succession. *PLOS ONE* **10**, e0123741 (2015).
6. Boukili, V. K. & Chazdon, R. L. Environmental filtering, local site factors and landscape context drive changes in functional trait composition during tropical forest succession. *Perspectives in Plant Ecology, Evolution and Systematics* **24**, 37–47 (2017).
7. Poorter, L. *et al.* Wet and dry tropical forests show opposite successional pathways in wood density but converge over time. *Nature Ecology & Evolution* **3**, 928–934 (2019).
8. Harvey, W. J. *et al.* The Legacy of Pre–Columbian Fire on the Pine–Oak Forests of Upland Guatemala. *Front. For. Glob. Change* **2**, (2019).
9. Lyon, S., Quesada-Pineda, H. & Smith, R. A Case Study to Determine Drivers and Barriers of Appalachian Forest Products in Central America. *Revista Forestal Mesoamericana Kurú* **9**, 40–50 (2012).
10. *Árboles de Centroamérica: un manual para extensionistas*. (CATIE, 2003).

11. Barbet-Massin, M., Jiguet, F., Albert, C. H. & Thuiller, W. Selecting pseudo-absences for species distribution models: how, where and how many?: How to use pseudo-absences in niche modelling? *Methods in Ecology and Evolution* **3**, 327–338 (2012).

12. Zurell, D. *et al.* A standard protocol for reporting species distribution models. *Ecography* (2020) doi:10.1111/ecog.04960.

13. Anderson, R. P. Harnessing the world's biodiversity data: promise and peril in ecological niche modeling of species distributions. *Annals of the New York Academy of Sciences* **1260**, 66–80 (2012).

14. Dupin, M. *et al.* Effects of the Training Dataset Characteristics on the Performance of Nine Species Distribution Models: Application to Diabrotica virgifera virgifera. *PLOS ONE* **6**, e20957 (2011).

15. Kriticos, D. J., Jarošik, V. & Ota, N. Extending the suite of bioclim variables: a proposed registry system and case study using principal components analysis. *Methods in Ecology and Evolution* **5**, 956–960 (2014).

16. Naimi, B. On uncertainty in species distribution modelling. (University of Twente, 2015). doi:10.3990/1.9789036538404.

17. Merow, C. *et al.* What do we gain from simplicity versus complexity in species distribution models? *Ecography* **37**, 1267–1281 (2014).

18. Fourcade, Y., Besnard, A. G. & Secondi, J. Paintings predict the distribution of species, or the challenge of selecting environmental predictors and evaluation statistics. *Global Ecol Biogeogr* **27**, 245–256 (2018).

19. Hidalgo, H. G. & Alfaro, E. J. Skill of CMIP5 climate models in reproducing 20th century basic climate features in Central America. *International Journal of Climatology* **35**, 3397–3421 (2015).

20. Knutti, R., Masson, D. & Gettelman, A. Climate model genealogy: Generation CMIP5 and how we got there. *Geophysical Research Letters* **40**, 1194–1199 (2013).

21. Gaspard, G., Kim, D. & Chun, Y. Residual spatial autocorrelation in macroecological and biogeographical modeling: a review. *Journal of Ecology and Environment* **43**, 19 (2019).

22. Diniz-Filho, J. A. F. *et al.* Partitioning and mapping uncertainties in ensembles of forecasts of species turnover under climate change. *Ecography* **32**, 897–906 (2009).

23. Watling, J. I. *et al.* Performance metrics and variance partitioning reveal sources of uncertainty in species distribution models. *Ecological Modelling* **309–310**, 48–59 (2015).

24. Warren, D. L., Matzke, N. J. & Iglesias, T. L. Evaluating presence-only species distribution models with discrimination accuracy is uninformative for many applications. *Journal of Biogeography* **47**, 167–180 (2020).

25. Calabrese, J. M., Certain, G., Kraan, C. & Dormann, C. F. Stacking species distribution models and adjusting bias by linking them to macroecological models. *Global Ecology and Biogeography* **23**, 99–112 (2014).

26. Schwarz, J. & Heider, D. GUESS: projecting machine learning scores to well-calibrated probability estimates for clinical decision-making. *Bioinformatics* **35**, 2458–2465 (2019).
